# Histone H3 deacetylation promotes host cell viability for efficient infection by *Listeria monocytogenes*

**DOI:** 10.1371/journal.ppat.1010173

**Published:** 2021-12-20

**Authors:** Matthew J. G. Eldridge, Mélanie A. Hamon

**Affiliations:** Pasteur, Chromatine et Infection G5, Paris, France; Boston Children’s Hospital, UNITED STATES

## Abstract

For many intracellular bacterial pathogens manipulating host cell survival is essential for maintaining their replicative niche, and is a common strategy used to promote infection. The bacterial pathogen *Listeria monocytogenes* is well known to hijack host machinery for its own benefit, such as targeting the host histone H3 for modification by SIRT2. However, by what means this modification benefits infection, as well as the molecular players involved, were unknown. Here we show that SIRT2 activity supports *Listeria* intracellular survival by maintaining genome integrity and host cell viability. This protective effect is dependent on H3K18 deacetylation, which safeguards the host genome by counteracting infection-induced DNA damage. Mechanistically, infection causes SIRT2 to interact with the nucleic acid binding protein TDP-43 and localise to genomic R-loops, where H3K18 deacetylation occurs. This work highlights novel functions of TDP-43 and R-loops during bacterial infection and identifies the mechanism through which *L*. *monocytogenes* co-opts SIRT2 to allow efficient infection.

## Author summary

To cause systemic disease *Listeria monocytogenes* assumes an intracellular lifestyle which supports its growth and dissemination during infection. In order to maintain the intracellular niche *L*. *monocytogenes* manipulates various host cell processes thereby promoting its own survival and infection. One such example is the hijacking of a host deacetylase called SIRT2 which upon infection localises to chromatin, specifically modifies lysine 18 of histone H3 and promotes intracellular bacterial growth. Here we identify how SIRT2 promotes infection. We show that SIRT2-mediated H3K18 deacetylation counteracts infection-induced DNA damage and identify the molecular complex at play. Such SIRT2 activity has a crucial role in promoting host cell viability during infection, allowing for better survival upon heavy intracellular bacterial burden, and resulting in enhanced infection by *L*. *monocytogenes*.

## Introduction

Intracellular parasitism requires a delicate balance between pathogen growth and the maintenance of the intracellular niche as premature death of the host cell would lead to inefficient infection. The Sirtuin family (SIRT1-7) of NAD^+^-dependent deacetylases are vital protective factors against numerous cellular stresses, and play key roles in many biological processes that maintain cellular homeostasis, including cell cycle, metabolism and DNA repair [1,2], however their roles during infection have not been well characterised.

Our previous work identified a novel function of Sirtuin 2 (SIRT2) during infection with the bacterial pathogen *Listeria monocytogenes* which causes SIRT2-nuclear accumulation. Upon infection SIRT2 becomes enriched on chromatin at the transcriptional start sites (TSSs) of certain genes and induces deacetylation of H3K18 independently of the cell cycle [3]. During infection, nuclear import of SIRT2, mediated in part by importin IPO7; and dephosphorylation of SIRT2 at serine 25 to permit chromatin binding, enable H3K18 deacetylation [4,5]. Importantly, SIRT2 activity at chromatin is essential for efficient *L*. *monocytogenes* infection in vitro and in vivo though how bacterial hijacking of SIRT2 promotes infection remains unknown.

Sirtuins have distinct subcellular localisations and diverse substrates, however all regulate DNA and chromatin to varying extents [1], particularly in response to genotoxic stress. Nuclear Sirtuins 1, 6 and 7 have the most clearly defined roles in maintaining genome integrity upon genotoxic stress. SIRT1 promotes DNA repair foci formation by deacetylating histones [6] and repair proteins such as KU70 [7], WRN [8] and XPA [9]. SIRT6 directly binds DNA breaks and promotes repair protein recruitment [10] and has been described to maintain the integrity of pericentric genomic regions through H3 lysine 18 (H3K18) deacetylation [11]. Similarly, SIRT7 promotes recruitment of the repair protein 53BP1 at sites of DNA damage, which also requires H3K18 deacetylation which enhances non-homologous end joining (NHEJ) [12]. SIRT7 does not bind damaged DNA and instead requires Poly [ADP-ribose] polymerase 1 (PARP1) to localise to double strand breaks [10,12]. By comparison, the mitochondrial Sirtuins have indirect roles in preserving DNA stability. SIRT3 protects mtDNA by limiting mitochondrial superoxide levels [13] and positively regulating the DNA repair protein OGG1 [14], while SIRT4 represses mitochondrial glutamine metabolism in response to genotoxic stress, thus promoting cell cycle arrest to allow for efficient DNA repair [15]. Sirtuin 2 (SIRT2) is unique as it is the only member to hold a predominantly cytoplasmic localisation and have clear regulatory roles across multiple subcellular compartments, functioning in metabolism, cell cycle, inflammation, and oxidative stress responses [2,16,17].

When localised to the nucleus, SIRT2 also functions to maintain genome stability. Like other Sirtuins these protective effects are brought about by deacetylating both histone and non-histone proteins, including the deacetylation of H4K16 [18], the anaphase-promoting complex/cyclosome (APC/C) [19] and the CDK9 kinase [20]. SIRT2-dependent regulation of these proteins ensures correct cell cycle progression and mitosis, and thus point to SIRT2 having essential roles in maintaining genome stability that are linked to its nuclear accumulation and association with chromatin.

During infection, *L*. *monocytogenes* induces DNA breaks in the host genome via an unknown mechanism which is independent of reactive oxygen species (ROS) [21,22]. Coupled to this *L*. *monocytogenes* also actively modulates the host DNA damage response (DDR) by triggering the degradation of the damage sensor MRE11, a process which enhances infection [22]. Given the roles of Sirtuins and H3K18 deacetylation in maintaining genome integrity, we hypothesised that SIRT2 could mitigate infection-induced genotoxic stress in order to better maintain the intracellular replicative niche [23–28].

In this study we show that SIRT2 activity protects host cells from DNA damage and promotes host cell survival during infection with *L*. *monocytogenes*. At the molecular level, we show that an interaction with the DNA/RNA binding protein TDP-43 is essential for SIRT2 enrichment at the transcription start site (TSS) of SIRT2-regulated genes and H3K18 deacetylation during infection. In fact, SIRT2 and TDP-43 function with genomic DNA:RNA hybrids called R-loops to reduce the accumulation of host DNA damage caused by infection. Therefore, we show that during infection, the activity of SIRT2 on H3K18 is key in regulating cellular health, which is exploited by *L*. *monocytogenes* to maintain host genome integrity and cell viability thereby promoting infection.

## Results

### SIRT2 activity maintains host cell viability during infection

**Fig 1:**
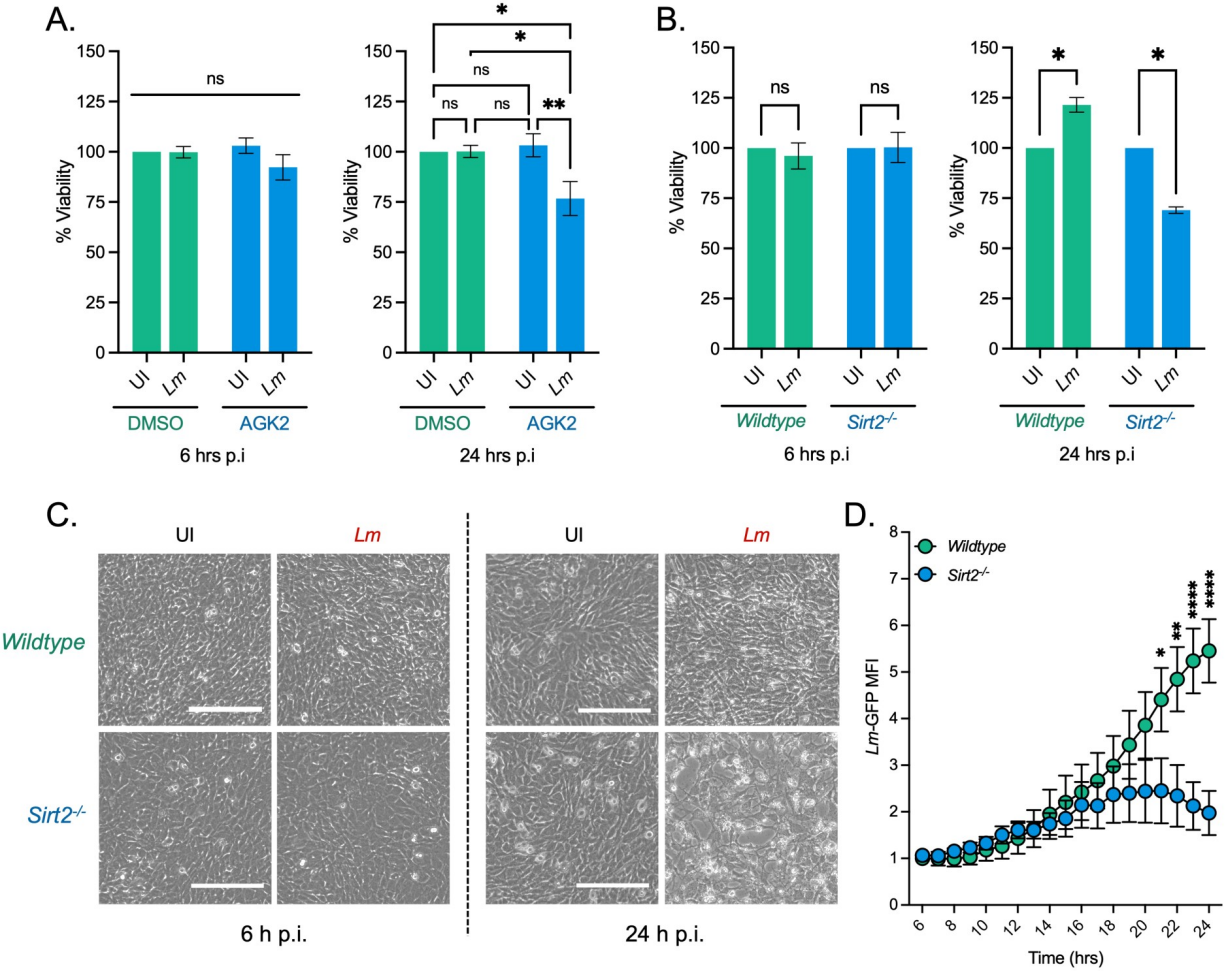
SIRT2 maintains host cell viability during *L*. *monocytogenes* infection. **(A)** Cell cytotoxicity measured by alamarBlue assay. HeLa cells pre-treated for 2 hours in DMSO or 5 mM AGK2 were left uninfected (UI) or infected (*Lm*) with *L*. *monocytogenes* for 6 and 24 hours. Results are expressed as percent viability of uninfected DMSO treated cells. Plot shows mean ± SEM from at least three independent experiments. Statistical significance was determined by a Kruskal-Wallis test (ns = not significant, * = *p* < 0.05, ** = *p* < 0.01). **(B)** Cell cytotoxicity measured by alamarBlue assay. MEFs from wildtype or *Sirt2*^*-/-*^ mice left uninfected (UI) or infected (*Lm*) with *L*. *monocytogenes* for 6 and 24 hours. Cytotoxicity was measured by alamarBlue assay. Results are expressed as percent viability of uninfected cells. Plot shows mean ± SEM from five independent experiments. Statistical significance was determined by a Kruskal-Wallis test (ns = not significant, * = *p* < 0.05). **(C)** Brightfield microscopy images of MEFs infected for 6 h and 24 h. Images taken at 10× magnification. Scale bar is 200 μm. **(D)** Mean fluorescence intensity of GFP-expressing *L*. *monocytogenes* (*Lm-GFP)* in infected wildtype and *Sirt2*^-/-^ MEFs (S1 Movie). Graph shows mean MFI relative to wildtype cells at 6 hrs ± SEM over time relative to 6 hr timepoint from four independent experiments. Statistical significance determined by two-way ANOVA with FDR Benjamini-Hochberg (BH) correction for multiple comparisons (** = *p* < 0.01, **** = *p* <0.0001).

We performed a cell viability assay cells using alamarBlue an oxidation-reduction indicator which undergoes fluorometric activation in response to cellular metabolic reduction. We measured alamarBlue fluorescence in uninfected and infected HeLa cells, with or without a SIRT2 inhibition. Interestingly, infection alone did not cause any reduction in host cell viability at either 6 or 24 hours post infection. However, in the presence of AGK2, a SIRT2 inhibitor, infected cells lose viability (Fig 1A). After 6 hours of infection, a slight 10% decrease in viability is detected in AGK2 treated cells, and by 24 hours a significant decrease of 30%, as compared with uninfected cells, is seen (Fig 1A). Importantly, AGK2 treatment alone did not lead to a decrease in cell viability (Fig 1A). Supporting this data, we performed cell counting assays at 6 h and 24 h post infection and were able to show that fewer intact cells were recovered at these time points (S1A Fig). Temporally, this reduction in host cell viability also coincides with a significant reduction in intracellular bacteria (S1B Fig).

As HeLa cells are carcinoma-derived with known aberrant regulation of cell viability, we performed similar alamarBlue assays in primary immortalised mouse embryonic fibroblasts (MEFs) derived from wildtype or *Sirt2*^*-/-*^ mice. Similar to HeLa cells wildtype and *Sirt2*^*-/-*^ MEFs display no decrease in cell viability at 6 hours post infection. However, by 24 hours infected *Sirt2*^*-/-*^ cells also display a significant reduction in cell viability (Fig 1B). Likewise, this corresponds with an observed decrease in intracellular *L*. *monocytogenes* (S1C Fig). Consistent with the alamarBlue assay microscopic analysis shows that by 24 hours post infection fewer *Sirt2*^*-/-*^ cells survive infection (Fig 1C). Therefore, although *L*. *monocytogenes* infection alone does not significantly impact cell viability, pharmacological inhibition, or loss SIRT2 activity during infection leads to significant cell death and reduced infection.

To better understand the dynamics of cell viability and how it impacts infection we performed time lapse microscopy using GFP-expressing *L*. *monocytogenes* (S1 Movie). Interestingly, infected *Sirt2*^-/-^ cells begin to show signs of cell death starting from ~12 hours post-infection which intensifies further up to 24 hours. Following the onset of cell death, we begin to detect a decrease in bacterial numbers and suggest it is the highly infected *Sirt2*^-/-^ cells which undergo death, and only cells with low levels of intracellular bacteria survive (S1D Fig). By comparison wildtype cells do not show evident signs of cell death and support higher levels of infection at 24 hours as detected by higher levels of GFP fluorescence. In agreement with CFU enumeration, quantification of *Lm*-GFP signal shows that during the early phases of infection both wildtype and *Sirt2*^-/-^ cells support bacterial growth up to ~13 hours post infection. However, following this time bacterial growth in *Sirt2*^-/-^ plateaus and then decreases after 20 hours of infection (Fig 1D and S1 Movie) which corresponds to the loss of host cells. Therefore, wildtype cells survive better and therefore support higher levels of infection (Fig 1D and S1 Movie).

### SIRT2 activity on H3K18 protects cells from infection-induced DNA damage

Given the role Sirtuins have in maintaining genome integrity upon stress, we examined the consequences of SIRT2 inhibition on DNA damage accumulation during infection. We monitored DNA damage by measuring the nuclear fluorescence intensity of the DNA damage marker γH2Ax during late infection, in the presence or absence of the SIRT2 inhibitor AGK2.

**Fig 2:**
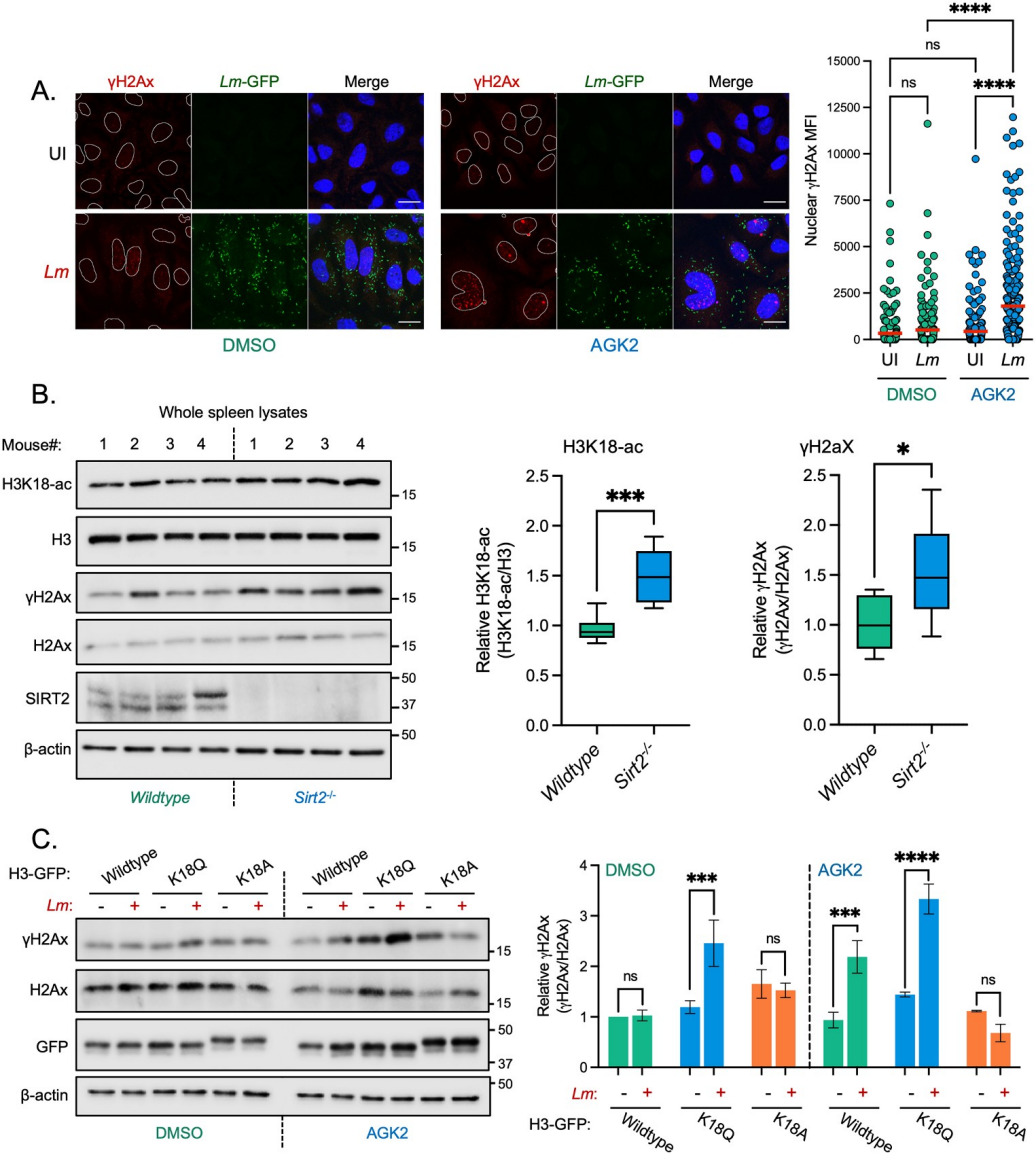
SIRT2 activity and H3K18 deacetylation protect host cells from DNA damage during *L*. *monocytogenes* infection. **(A)** Representative images of immunofluorescence (left) detection of endogenous γH2Ax (red) in HeLa cells left uninfected (UI) or infected for 24 hours with GFP-expressing *L*. *monocytogenes* (*Lm*-GFP). Scale bar is 20 μm. Quantification of nuclear γH2Ax (right) from HeLa cells, data points represent the mean fluorescence intensity (MFI) of γH2Ax within individual nuclei. Graphs display quantification from 2 independent experiments with the mean values of each condition are represented by a red line. Statistical significance was determined by one-way ANOVA with FDR Benjamini-Hochberg (BH) correction for multiple comparisons (ns = not significant, **** = *p* < <0.0001). **(B)** Immunoblot detection of stated proteins from infected mouse spleen lysates (left). Quantification of normalised H3K18-ac and γH2Ax levels. Graphs show collated values from 8 mice from two independent experiments, box and whisker plot with solid line denoting the median value. Statistical significance was determined by Two-tailed Unpaired t test (* = *p* < 0.05, *** = *p* <0.001). **(C)** Immunoblot detection of stated proteins (left) from whole cell lysates of HeLa cells left uninfected (-) or infected with *L*. *monocytogenes* (+) for 24 hours. Cells are expressing stated H3-GFP plasmids and treated with DMSO or 5 mM AGK2. Images are representative of three independent experiments. Quantification of γH2Ax levels (right) relative to uninfected. Results are expressed as intensity of actin normalised γH2Ax bands relative to actin normalised total H2Ax. Graph shows the mean ± SEM from three independent experiments. Statistical significance was determined by one-way ANOVA with Fisher’s LSD test (ns = not significant, *** = *p* <0.001, **** = *p* <0.0001).

Consistent with previous reports, *L*. *monocytogenes* infection induces basal levels of DNA damage illustrated by a slight increase in γH2Ax levels in host cell nuclei (Fig 2A). At 24 hours post-infection we observed a 15% increase in the number of γH2Ax positive cells as compared with uninfected conditions, accompanied by ~1.5-fold increase in γH2Ax mean fluorescence intensity (MFI) across the cell population (Fig 2A and S2A Fig). In uninfected cells treated with AGK2 there was no significant increase in γH2Ax staining, suggesting that under resting conditions SIRT2 has no significant effect on the induction of DNA damage. By contrast, infected AGK2-treated cells accumulated significantly higher levels of DNA damage by 24 hours post infection, as evidenced by a 35% increase in the number of γH2Ax positive cells and a concurrent ~4-fold increase in the average nuclear γH2Ax MFI (Fig 2A and S2A Fig). By western blot γH2Ax levels increase following infection in an MOI-dependent manner, but only in the presence of AGK2 (S2B Fig). Similar results were also obtained in MEFs where infected *Sirt2*^-/-^ cells have significantly elevated levels of γH2Ax as compared with wildtype and uninfected controls (S2C Fig). These data indicate that SIRT2 activity suppresses the accumulation of DNA damage during infection.

Because SIRT2 also regulates cell cycle progression, we investigated whether the observed differences in DNA damage might be linked to cell cycle dysregulation. We assessed the relative proportions of uninfected and infected HeLa cells in different cycle phases with or without SIRT2 inhibition. 6 hours post-infection cells exposed to *L*. *monocytogenes* show a small ~5% increase in the proportion of cells in G2/M however these differences are no longer present at 24 hours post infection when the highest levels of γH2Ax are detected, suggesting that host cell cycle does not play a significant role in the accumulation of DNA damage and loss of cell viability observed during infection (S3 Fig).

The impact of SIRT2 on infection-induced DNA damage was also determined in vivo. Spleens from wildtype and *Sirt2*^-/-^ mice were collected 72 hours after intravenous infection with *L*. *monocytogenes* and levels of γH2Ax were assessed by immunoblotting. As previously reported, spleens from infected *Sirt2*^-/-^ mice had significantly higher levels of H3K18-ac and showed a trend towards lower bacterial numbers compared with wildtype mice (Fig 2B and S4B Fig) whilst acetylation levels from uninfected spleens showed no difference (S4B Fig). Strikingly levels of γH2Ax were significantly higher in infected *Sirt2*^-/-^ mice similar to what is observed in vitro, with no differences observed in uninfected conditions (Fig 2B and S4B Fig). Therefore, DNA damage also accumulates in vivo within organs that are targeted during infection in a *Sirt2*^-/-^ background.

Since SIRT2 targets many proteins in the cell, we asked whether the activity of SIRT2 towards H3K18 specifically responsible for suppressing DNA damage. To answer this question, we infected cells overexpressing GFP-tagged wildtype histone H3, or mutants where K18 was substituted with either glutamine (K18Q) or alanine (K18A) which respectively mimic acetylated and deacetylated H3K18. Under these conditions, DNA damage was measured by γH2Ax immunoblotting. Upon transfection and expression of wildtype H3, DNA damage is observed only in infected cells that were AGK2-treated (Fig 2C), similarly to what is observed by immunofluorescence. Alone, the expression of either mutant H3 K18Q or H3 K18A did not induce any significant increase in γH2Ax levels in resting cells. Strikingly though, upon infection, expression of H3 K18Q (DMSO treated) is sufficient to induce higher levels of γH2Ax (Fig 2C), similar to the levels induced by infection with combined AGK2 treatment. By contrast, expression of H3K18A does not increase γH2Ax upon infection and, in fact, blocks γH2Ax accumulation observed in AGK2 treated cells. Therefore, overexpression of H3 mutants which mimic histone acetylation is sufficient to cause DNA damage accumulation in infected cells. Consistently, expression of a deacetylated mimic is sufficient to protect cells from DNA damage, in the absence of SIRT2 activity.

Similar results were obtained upon expression of mutant H3 and assessing infection efficiency. We used western blotting to assess the intracellular levels of InlC, a listerial effector protein which is expressed when bacteria are intracellular and has been used as a readout of infection [22,29]. Consistent with our CFU data, cells expressing WT H3 show significantly reduced levels of InlC 24 hours post infection when treated with AGK2 (S4C Fig) indicating a stunted infection when SIRT2 is inhibited. On the contrary cells expressing H3 K18Q have lower InlC levels which are not significantly reduced further by AGK2 treatment. However, cells expressing H3 K18A show no change in InlC suggesting that H3K18 deacetylation can restore *L*. *monocytogenes* infection in SIRT2 inhibited cells.

Together, all these results suggest that SIRT2 activity towards H3K18 has a direct protective role against the accumulation of excessive DNA damage and is required to promote infection.

### SIRT2 interacts with TDP-43 for recruitment to chromatin

Although SIRT2 lacks intrinsic DNA binding capabilities, our previously published SIRT2 interactome found many putative interactors to be DNA binding proteins [5]. As such, we analysed this list to identify potential interacting partners which could anchor SIRT2 to DNA. Our previous work showed that SIRT2 was recruited to the TSSs of a subset of genes which are repressed during infection. We therefore used the GeneCards database, to compile lists of proteins known to interact with the TSSs of 5 different SIRT2-regulated genes (*MYLIP*, *ERCC5*, *LEF1*, *SYDE2*, *EHHADH*) and compared these against the SIRT2 interactome to identify common proteins. From this analysis only one SIRT2-putative interactor was found in common across all lists, TDP-43 (encoded by *TARDBP* gene) a DNA/RNA binding protein (S5 Fig). Further in silico analysis of previously identified infection-dependent SIRT2-repressed genes [3] showed that 72% of these have TDP-43 present at their TSSs by ChIP-seq (ENCODE portal). Therefore TDP-43 represented a suitable candidate protein to recruit SIRT2 to chromatin at specific loci during *L*. *monocytogenes* infection.

**Fig 3:**
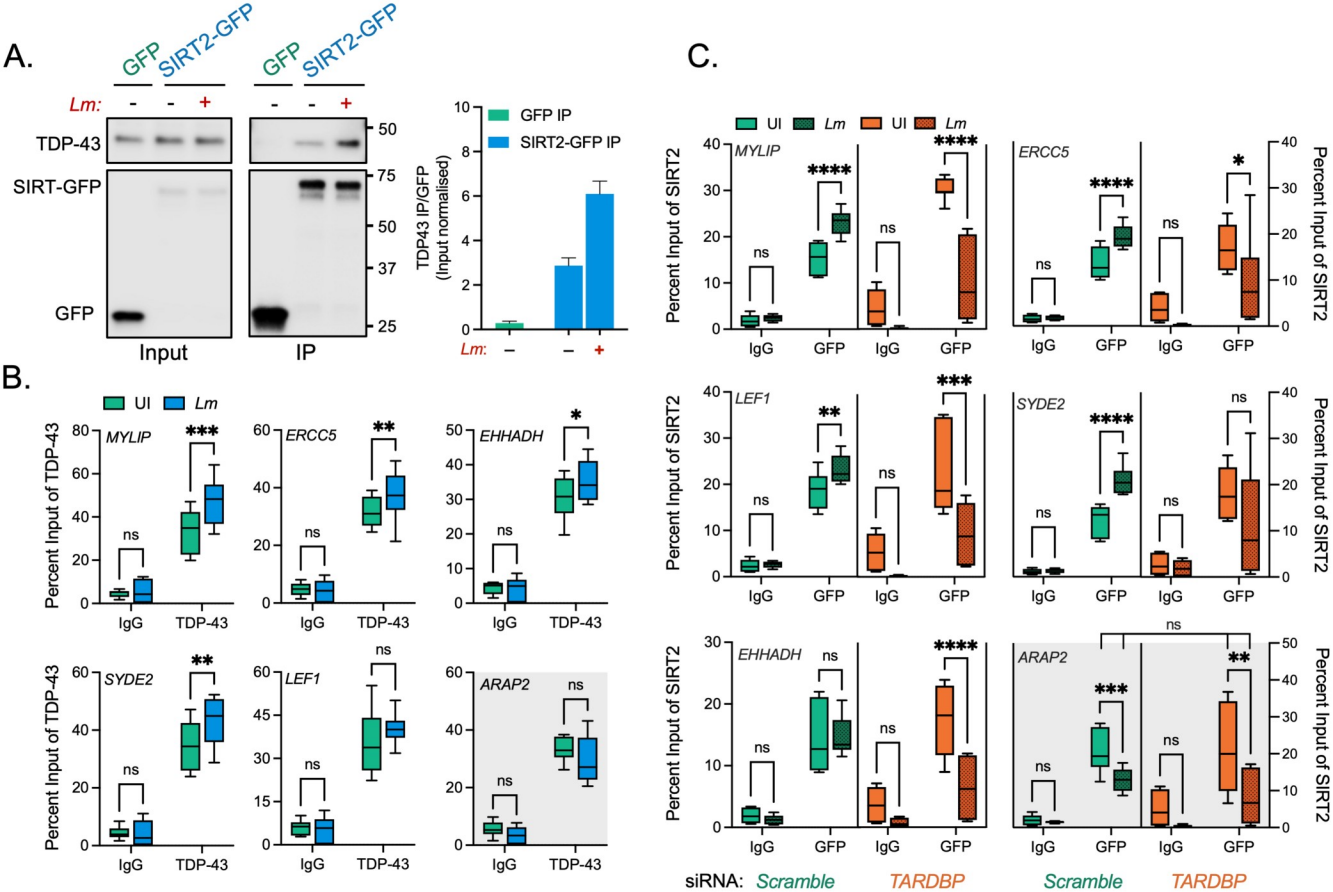
TDP-43 interacts with SIRT2 and is required for chromatin interactions and gene targeting upon infection. **(A)** HeLa cells expressing either GFP alone or SIRT2-GFP were left uninfected (-) or infected (+) for 3 hours. Cells were lysed and underwent immunoprecipitation using GFP-Trap agarose beads. Cell lysates (Input) and IP fractions were immunoblotted using antibodies against GFP or TDP-43 (left). Quantification of endogenous TDP-43 enriched by GFP or SIRT2-GFP (right). Graph shows input normalised intensities of TDP-43 protein relative to SIRT2-GFP intensity detected from the same sample. Enrichment is expressed relative to basal interaction observed on uninfected cells. Graph shows the mean ± SEM from three independent experiments. **(B)** Chromatin immunoprecipitation (ChIP) using non-targeting control (Ctrl IgG) or TDP-43 (TDP-43 IgG) targeting antibodies quantified by qPCR. Chromatin was extracted from uninfected (UI—green) or infected (EGD—blue) HeLa cells 6 hours post infection. qPCR was carried out using primers targeting the transcriptional start sites of stated SIRT2-dependent or independent (gray background) genes. Graphs show collated technical readings (n = 4) from three independent experiments and are presented as percent recovery of ChIP relative to input and plotted as box and whisker plot with solid line denoting the median value. Statistical significance determined by two-way ANOVA with FDR Benjamini-Hochberg (BH) correction for multiple comparisons (ns = not significant, * = *p* < 0.05, ** = *p* < 0.01, *** = *p* <0.001). **(C)** Chromatin immunoprecipitation (ChIP) using non-targeting control (Ctrl IgG) or GFP (GFP IgG) targeting antibodies quantified by qPCR. Chromatin was extracted from HeLa cells stably expressing SIRT2-GFP and transfected with non-targeting *Scramble* (Green) or *TARDBP* targeting (Orange) siRNA. Cells were left uninfected (UI -clear) or infected (EGD—dotted) for 6 hours. qPCR was carried out using primers targeting the transcriptional start sites of stated SIRT2-dependent or independent (red box) genes. Graphs show collated technical readings (n = 4) from three independent experiments and are presented as percent recovery of ChIP relative to input and plotted as box and whisker plot with solid line denoting the median value. Statistical significance determined by two-way ANOVA with FDR Benjamini-Hochberg (BH) correction for multiple comparisons (ns = not significant, * = *p* < 0.05, ** = *p* < 0.01, *** = *p* <0.001, **** = *p* <0.0001).

Interaction of SIRT2 with TDP-43 upon infection was determined by cell fractionation and immunoprecipitation, HeLa cells were transfected with plasmids encoding GFP alone or GFP-tagged SIRT2 (SIRT2-GFP), then left uninfected or infected with *L*. *monocytogenes* followed by immunoprecipitation from isolated nuclei. Immunoblotting analysis showed that endogenous nuclear TDP-43 co-precipitates with SIRT2-GFP but not GFP alone in uninfected cells (Fig 3A). Interestingly, following infection, TDP-43 binding to SIRT2 is further enriched by approximately 2-fold (Fig 3A). Consistent with our previous interactome analysis these data show that a basal interaction between SIRT2 and TDP-43 occurs in the nuclei of uninfected cells, and we show that this interaction is significantly enhanced in response to *L*. *monocytogenes* infection.

We previously identified Ser25 as a residue on SIRT2 that was dephosphorylated upon infection and was necessary for SIRT2 to become enriched at chromatin [4]. To address whether this post-translational modification could be involved in regulating the interaction between SIRT2 and TDP-43 we co-transfected HeLa cells with mCherry-TDP-43 and either WT SIRT2-GFP, phosphomimetic S25E SIRT2-GFP, or dephosphomimetic S25A SIRT2-GFP. Immunoprecipitation of TDP-43 with RFP-Trap beads followed by immunoblotting showed that all SIRT2 variants could interact with TDP-43. Furthermore, both SIRT2 variants displayed an augmented interaction with TDP-43, particularly the S25A dephosphomimetic displayed a ~2.5-fold increase in its interaction as compared with the WT variant. This increase is similar to that observed following infection, suggesting that S25 dephosphorylation has a role in regulating this interface between SIRT2 and TDP-43 (S6 Fig).

We further wanted to establish whether TDP-43 was required for SIRT2-binding to DNA. Chromatin immunoprecipitation PCR (ChIP-PCR) of endogenous TDP-43 from uninfected HeLa cells showed that TDP-43 localises to the TSSs of SIRT2-regulated genes *MYLIP*, *ERRC5*, *LEF1*, *SYDE2* and *EHHADH*, consistent with multiple ChIP-seq data sets available from the ENCODE project database [30]. Following *L*. *monocytogenes* infection, TDP-43 shows a slight, and in most cases significant, enrichment of ~10% at these genetic loci (Fig 3B). By comparison *ARAP2*, a SIRT2 independent gene, does not show TDP-43 recruitment upon infection. To determine whether TDP-43 is necessary for the recruitment of SIRT2 to chromatin, we performed ChIP-PCR of GFP tagged SIRT2 from uninfected and infected cells transfected with either scramble or TDP-43 (*TARDBP*) targeting siRNA (S7A Fig). For all genes tested, knockdown of TDP-43 does not change the basal level of SIRT2 recruitment in uninfected cells (Fig 3C). As previously demonstrated, infection causes significant recruitment of SIRT2 to the TSSs of *MYLIP*, *ERRC5*, *LEF1*, *SYDE2* and *EHHADH* but not *ARAP2*. However, loss of TDP-43 from cells blocks recruitment of SIRT2, and in fact significantly reduces SIRT2 levels at the TSSs of these genes by ~5–15%. By contrast, the SIRT2 activity-independent gene *ARAP2* shows a decrease in SIRT2 enrichment during infection which is not altered by the loss of TDP-43 (Fig 3C). These data show that whilst TDP-43 is not required for the basal localisation of SIRT2 to chromatin in resting cells, the specific interaction and enrichment of SIRT2 which occurs following infection is dependent on TDP-43, and loss of TDP-43 dysregulates SIRT2-chromatin dynamics (Fig 3C). Taken together these data show that infection enhances the interaction between SIRT2 and TDP-43 in the nucleus, and that TDP-43 is necessary for the infection-induced enrichment of SIRT2 at the chromatin level to specific genetic locations.

### TDP-43 is required for SIRT2-dependent functions during infection

**Fig 4:**
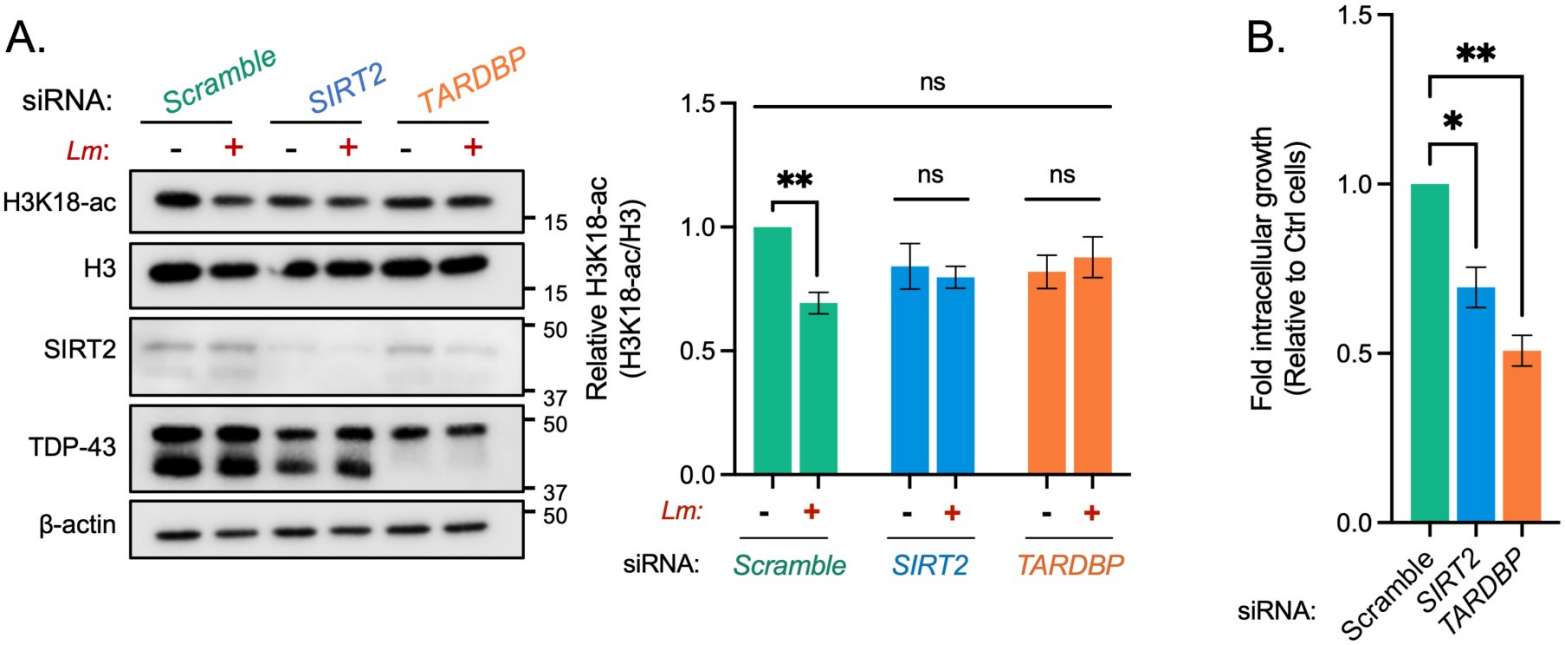
Silencing TDP-43 expression blocks H3K18 deacetylation and other SIRT2-related phenotypes during infection. **(A)** Representative image of H3K18 acetylation, SIRT2 and TDP-43 levels detected by immunoblotting (left) 6 hours post infection in uninfected HeLa (−) and *L*. *monocytogenes*–infected cells (+) transfected with stated siRNA. Quantification of H3K18 acetylation levels (right): band intensity of H3K18-ac and total H3 levels are normalised to β-actin followed by normalisation of H3K18-ac to total H3. Values are expressed as normalised band intensity relative to uninfected *Scramble* cells. Error bars represent SEM of four independent experiments. Statistical significance was determined by a Kruskal-Wallis test (ns = not significant, ** = *p* < 0.01). **(B)** Fold change of intracellular *L*. *monocytogenes* colony forming units during infection of HeLa cells transfected with stated siRNAs. Data are presented as fold-change in recovered intracellular CFU between 2.5 and 24 hours post infection relative to *Scramble* siRNA cells. Graph shows the mean ± SEM from three independent experiments. Statistical significance was determined by a Kruskal-Wallis test (* = *p* < 0.05, ** = *p* < 0.01).

In the context of *L*. *monocytogenes* infection, our data strongly suggests that TDP-43 acts as a scaffold for SIRT2 recruitment to specific gene loci, and therefore would be essential for enabling SIRT2-dependent processes and related downstream phenotypes. 48 hours prior to infection HeLa cells were transfected with scrambled siRNA or a pool of three siRNAs which target either *SIRT2* or *TARDBP* mRNA, reducing their respective levels by ~70% and 90% (S7B and S7C Fig). As expected, in HeLa cells transfected with scramble siRNA, H3K18 deacetylation occurred normally during infection. Global H3K18-ac levels decreased by 30–40% as compared with uninfected cells (Fig 4A). However, this decrease in acetylation levels was blocked in *TARDBP* silenced cells similarly to what was observed upon *SIRT2* silencing (Fig 4A). Therefore, TDP-43 is required for SIRT2-dependent H3K18 deacetylation during infection.

Since blocking SIRT2 activity results in lower intracellular bacteria numbers, we performed similar experiments upon silencing of TDP-43 to assess *L*. *monocytogenes* replication/survival. Similarly to what is observed upon blocking SIRT2, TDP-43 silencing also causes a reduction in bacterial numbers 24-hours post infection, where 50% fewer bacteria were recovered relative to scramble controls (Fig 4B). Therefore, these results show that, like SIRT2, loss of TDP-43 has a negative impact on bacterial replication/survival within host cells and is therefore required to promote infection. Altogether, our data demonstrate that TDP-43 is required for the execution of SIRT2-dependent H3K18 deacetylation during infection, and for the advantage SIRT2 can confer to *L*. *monocytogenes* during infection.

### R-loops are required for infection induced H3K18 deacetylation

**Fig 5:**
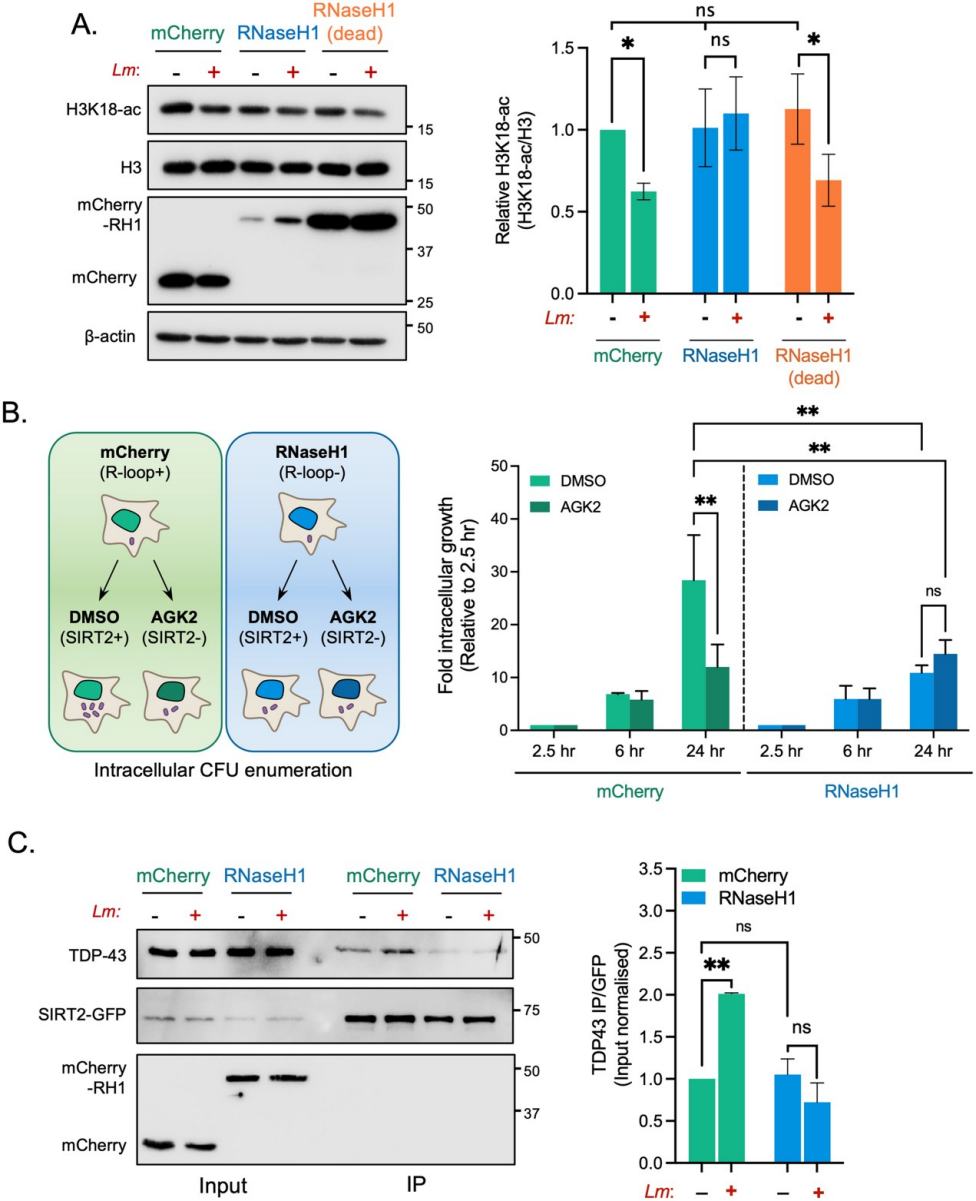
Blocking R-loop formation by overexpressing RNaseH1 inhibits infection induced H3K18 deacetylation and supresses bacterial intracellular survival. **(A)** Immunoblot analysis of H3K18 acetylation 6 hours post infection (left) in uninfected(-) and infected (+) HeLa cells expressing either mCherry, WT RNaseH1 or catalytically inactive RNaseH1 (dead). Quantification of H3K18 acetylation (right). H3K18-ac and total H3 levels band intensities are normalised to β-actin followed by normalisation of H3K18-ac to total H3. Values are expressed as normalised band intensity relative to uninfected mCherry cells. Error bars represent the SEM from at least three independent experiments. Statistical significance was determined by a Kruskal-Wallis test (ns = not significant, * = *p* < 0.05). **(B)** Schematic (left) outlining experimental procedure of experiment. Fold change of intracellular *L*. *monocytogenes* colony forming units (right) during infection of HeLa cells expressing stated plasmid constructs treated with either DMSO or 5 mM AGK2. Data are presented as the fold-change in recovered intracellular CFU for each cell type at 6 and 24 hours post infection relative to their corresponding 2.5-hour time point. Graphs show the mean ± SEM from three independent experiments. Statistical significance was determined by two-way ANOVA with FDR Benjamini-Hochberg (BH) correction for multiple comparisons (ns = not significant, ** = *p* < 0.01). **(C)** HeLa cells stably expressing SIRT2-GFP were transfected with plasmids expressing mCherry or WT RNaseH1 were left uninfected (-) or infected (+) for 3 hours. Cells were lysed and underwent immunoprecipitation using GFP-Trap agarose beads. Cell lysates (Input) and IP fractions were immunoblotted using stated antibodies. Graph (right) shows quantification of TDP-43 enriched by SIRT2-GFP. Data expressed as input normalised band intensity of TDP-43 relative to SIRT2-GFP intensity from the same sample. Graph shows the mean ± SEM from three independent experiments. Statistical significance was determined by one-way ANOVA with FDR Benjamini-Hochberg (BH) correction for multiple comparisons (ns = not significant, ** = *p* < <0.005, *** = *p* < <0.001).

TDP-43 is a nuclear DNA/RNA binding protein that specifically recognises single stranded nucleic acids. Recently, TDP-43 has been shown to interact with nucleic acid structures called R-loops, which preferentially form at TSSs when newly transcribed RNA anneals to the coding strand of DNA forming an RNA:DNA hybrid and displaces a strand of ssDNA. Since no straightforward method exists to detect R-loops the most common way to assess their function is though overexpression of RNaseH1 which resolves DNA/RNA hybrids. Cells were transfected either with a mCherry control plasmid (pICE-mCherry-NLS) or a RNaseH1 expressing plasmid (pICE-RNaseH1-WT-NLS), and H3K18 deacetylation was monitored by immunoblotting. Expression of the control mCherry plasmid had no effect on the previously observed infection induced H3K18 deacetylation. However, cells overexpressing RNaseH1 displayed no difference in acetylation levels, demonstrating that RNaseH1 expression blocks infection-induced deacetylation. In contrast, cells transfected with a catalytically inactive mutant of RNaseH1 (pICE-RNaseH1-D10R, E48R-NLS) retain the ability to deacetylate H3K18 upon infection (Fig 5A), which demonstrates that only catalytically active RNaseH1 blocks H3K18 deacetylation. These data therefore suggest that resolving of R-loops by expression of RNaseH1 blocks histone deacetylation, thereby indicating that the presence or formation of R-loops is required for this modification to occur.

Similarly, we overexpressed RNaseH1 to determine whether resolving R-loops would influence the intracellular survival of *L*. *monocytogenes* as observed upon loss of SIRT2 or TDP-43 (Fig 4B). In agreement with results from S1B Fig, cells transfected with a control mCherry plasmid have reduced intracellular CFU when treated with AGK2. By comparison, overexpression of RNaseH1 alone is sufficient to cause the same decrease in recovered bacterial colonies 24 hr post infection with no impact on infection at 6h (Fig 5B and S8 Fig). Interestingly, additional inhibition of SIRT2 with AGK2 does not have a cumulative effect on intracellular bacterial numbers, suggesting that R-loops and SIRT2 are regulating infection through the same pathway (Fig 5B).

With these results we hypothesised that R-loops may serve as a platform for the recruitment of TDP-43 and SIRT2. We performed immunoprecipitation experiments for SIRT2 and TDP-43 from cells expressing mCherry or RNaseH1. In cells expressing mCherry alone we observed an ~2-fold increase in TDP-43 enrichment by SIRT2 following infection (Fig 5C), similar to results shown in Fig 2A. On the contrary, in cells overexpressing RNaseH1, which lack R-loops, the infection-induced interaction between SIRT2 and TDP-43 is inhibited (Fig 5C) suggesting that R-loops are required for SIRT2:TDP-43 complex formation in response to infection.

Together these data establish that R-loops are required for infection-induced H3K18 deacetylation, and that promoting R-loop resolution by overexpression of RNaseH1 alone is sufficient to negatively affect the survival of *L*. *monocytogenes* in host cells, phenotypically copying the loss of SIRT2 and TDP-43. This effect seems to function at the level of SIRT2:TDP-43 complex formation.

### TDP-43 and R-loops are required to protect against excessive infection induced DNA damage

**Fig 6:**
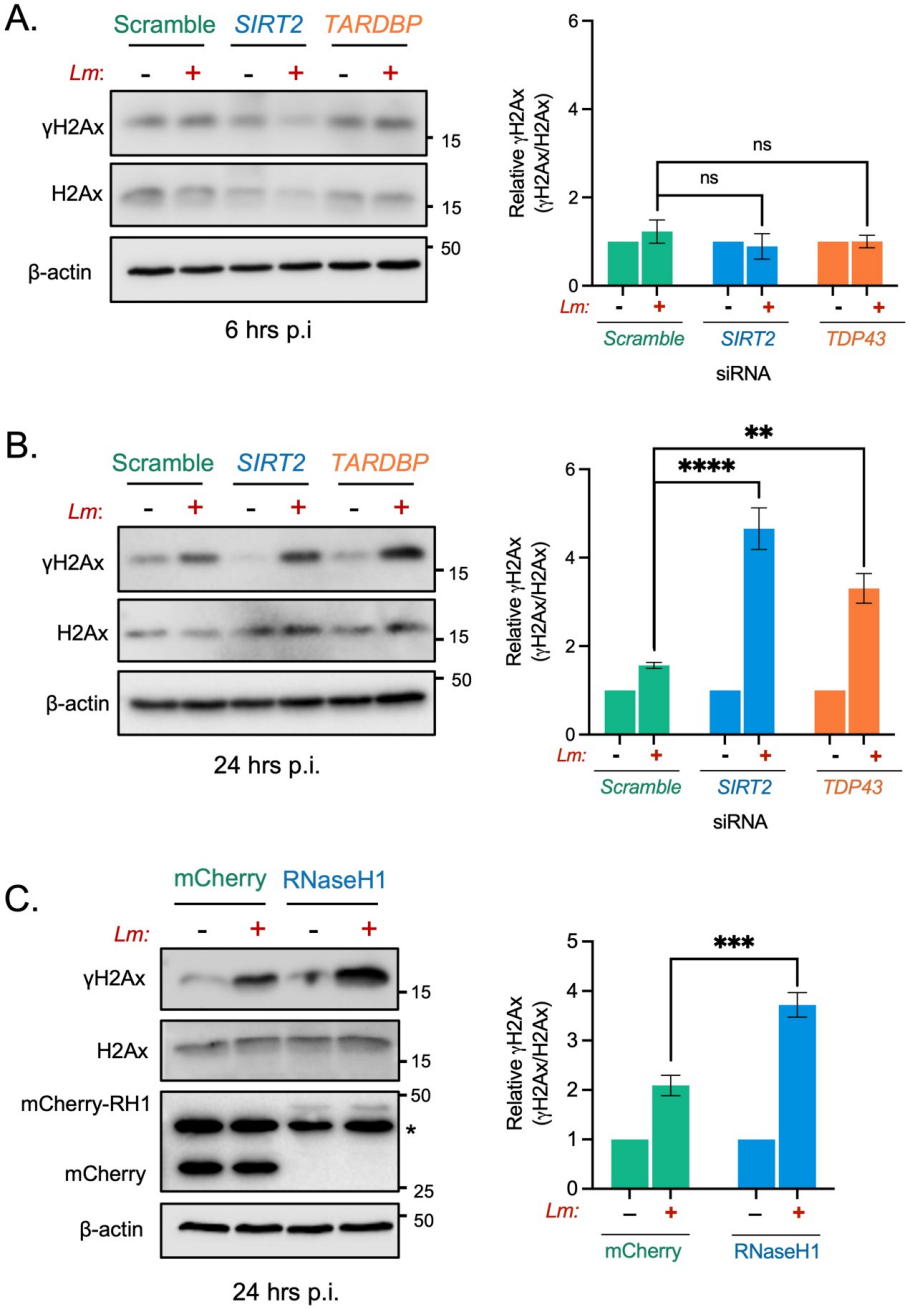
SIRT2, TDP-43 and R-loops function to protect cells from infection induced DNA damage. Immunoblot detection of γH2Ax and total H2Ax from whole cell lysates of HeLa cells transfected with stated siRNAs and left uninfected (-) or infected (+) for **(A)** 6 hours or **(B)** 24 hours. Quantified band intensities of γH2Ax levels are present in graphs (right). Results are expressed as intensity of actin normalised γH2Ax bands relative to actin normalised total H2Ax. Graph shows the mean ± SEM from at least three independent experiments. Statistical significance was determined by one-way ANOVA with FDR Benjamini-Hochberg (BH) correction for multiple comparisons (ns = not significant, ** = *p* <0.01, **** = *p* <0.0001). **(C)** HeLa cells expressing either mCherry or mCherry-RNaseH1 were infected for 24 hours, immunoblot analyses and quantification of γH2Ax (right) performed as stated for (A) and (B) *denotes non-specific band. Graph shows the mean ± SEM from five independent experiments. Statistical significance was determined by Two-tailed Unpaired t test (*** = *p* <0.001).

Our data shows that SIRT2 activity and H3K18 deacetylation reduce the genotoxic effects of *L*. *monocytogenes* infection. We therefore asked whether TDP-43 and R-loops, which are also required for infection induced H3K18 deacetylation, would impact the accumulation of DNA damage. At earlier timepoints, where no infection-induced γH2Ax is observed, loss of either SIRT2 or TDP-43 does not result in heightened γH2Ax in cells (Fig 6A). Consistent with our results using AGK2, infected cells depleted of SIRT2 or TDP-43 by RNAi display significantly elevated levels of γH2Ax in infected cells, as detected by western blot at 24 hours post infection (Fig 6B). Likewise, blocking the formation of R-loops by overexpressing RNaseH1 also significantly increased the amount of γH2Ax detected in infected cells at 24 hours post infection (Fig 6C). Therefore, R-loops are required to protect infected cells from excessive DNA damage and are important for a productive *L*. *monocytogenes* infection.

## Discussion

**Fig 7:**
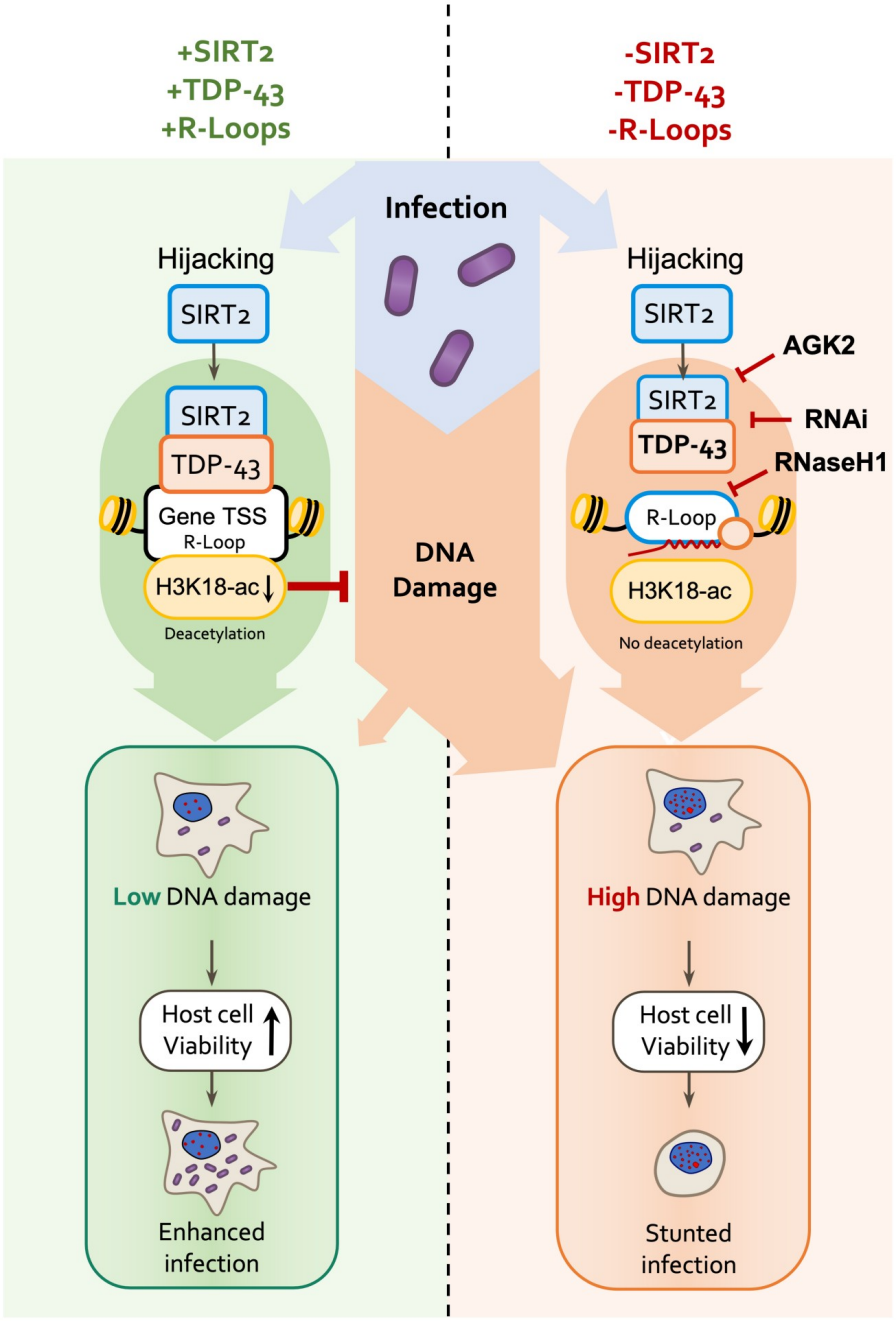
Model of host genome integrity protection by SIRT2. Schematic model of SIRT2 mechanisms of host cell protection and enhancement of infection. During infection, SIRT2 activity is hijacked by *Listeria* and is translocated to nucleus mediating H3K18 deacetylation. Recruitment to chromatin and histone deacetylation require TDP-43 and R-loops, which define the localisation of SIRT2 to specific genes. H3K18 deacetylation then directly functions to protect host genomic DNA from accumulating excessive DNA damage induced during infection by unknown mechanisms. This promotes genome integrity and cell viability thereby better supporting the intracellular lifestyle of *Listeria* and resulting in enhanced infection.

For many intracellular pathogens promotion or maintenance of host cell survival is crucial for establishing an efficient intracellular replicative niche which promotes infection. For *L*. *monocytogenes* we show this is in fact the case and that SIRT2, TDP-43 and R-loops are necessary. Interestingly, without activation of this signalling axis host cells accumulate high levels of DNA damage during infection, lose viability and *Listeria* infection is restricted (Fig 7).

Previous studies have shown that *L*. *monocytogenes* generates DNA damage in the host independently of ROS through an unknown mechanism [21,22]. In addition, the *L*. *monocytogenes* toxin LLO also acts to dampen the host DDR and promotes infection by triggering the degradation of the host DNA damage sensor MRE11 [22]. Other work has suggested that such infection-induced DNA damage promotes infection by delaying host cell cycle progression and increasing the host cellular nucleotide pool which can be scavenged by bacteria, promoting their replication [21]. In macrophages, *L*. *monocytogenes* infection also induces DNA breaks which are generated by nitric oxide production in responses to TLR signalling which in turn activates a DDR pathway that regulates a pro-inflammatory transcriptional program to augment macrophage responses [31]. Consistent with its potential role in DDR during infection, our previously published SIRT2 interactome identified many DNA damage sensor and repair proteins including KU70/KU80, RPA1, FEN1 and PARP1 [5].

The results presented here define H3K18 deacetylation by SIRT2 as the key factor required for protection from DNA damage during infection. Others have shown H3K18 deacetylation by SIRT6 or SIRT7 similarly protects the genome by respectively silencing pericentric satellite DNA expression and promoting recruitment of the DNA repair protein 53BP1 [11,12]. Taken together, these suggest that H3K18 deacetylation serves as a mark of DNA damage upon cellular stress for the recruitment of repair proteins. The general role of this mark in cellular stress and infection will need to be investigated further.

TDP-43 is a ubiquitously expressed protein belonging to the heterogenous nuclear ribonucleoprotein (hnRNP) family which has specificity for single stranded TG/UG-rich DNA and RNA [32–35]. As such TDP-43 has been reported to regulate various aspects of RNA processing, for instance controlling exon skipping in transcripts which contain intronic UG-repeats such as *CFTR* [36]. Furthermore, though primarily nuclear, TDP-43 does function in the cytosol as part of RNA transporting granules, and is described to regulate the subcellular localization, translation and stability of mRNA [37,38]. TDP-43 is also appreciated to act as a direct transcriptional repressor, suppressing expression of chromosome integrated TAR DNA from HIV-1 [39], and the mouse gene *Acrv1* [40] though the precise mechanisms are unclear. Our results generated by ChIP-PCR of TDP-43 strongly suggest that during infection with *L*. *monocytogenes* TDP-43 binds DNA rather than RNA.

Though primarily monomeric, physiological oligomerisation of TDP-43 has also been described and is believed to regulate DNA-binding and stress resistance [41,42]. TDP-43 has been identified as a causative factor of the neurodegenerative disease amyotrophic lateral sclerosis (ALS), mostly commonly due to mutations which cause it to mislocalise to the cytoplasm and self-assemble into large prion-like aggregates [43]. As well as their direct pathological roles, ALS-related mutations also disrupt the native functions of TDP-43 revealing that it acts as a scaffold for the recruitment of DNA repair proteins. As such, ALS mutant or TDP-43 deficient neuronal cells have defects in NHEJ DNA repair and are more sensitive to genotoxic agents [44,45]. Our work suggests that, in the context of *L*. *monocytogenes* infection, TDP-43 recruits SIRT2 to chromatin, as it can for other DDR factors [44,45] and suggests a broader role of SIRT2 in other pathological contexts, perhaps in regulating DNA repair in ALS or other neurodegenerative illnesses.

R-loops are an emerging feature of chromatin which regulate processes such as DNA damage, but have not been studied in the context of infection. Whether they are detrimental or beneficial for the maintenance of genome integrity remains controversial [46–48]. Persistent R-loops have been demonstrated to cause DNA damage due to incorrect processing by nucleotide excision repair nucleases XPG and XPA or by blocking replication fork progression, resulting in the formation of double strand breaks [49,50]. However, R-loops can also function to promote DNA repair, particularly in the context of transcriptionally coupled homologous recombination repair and NHEJ [46,51,52]. During infection however, we find that blocking R-loop formation by overexpressing RNaseH1 leads to higher levels host DNA damage in response to *L*. *monocytogenes*, suggesting that R-loops play a protective role in this context.

In our previous work we demonstrated that SIRT2 targeted genes are also transcriptionally repressed during infection in a SIRT2 activity-dependent manner. Transcriptional repression is also a feature of the DDR as transcriptional machinery can interfere with DNA repair. Interestingly, in silico analysis shows that many SIRT2 regulated sequences contain or are predicted to contain R-loops; additionally there are multiple studies which demonstrate that TDP-43 localises to and interacts with R-loops [53–55]. We also show that TDP-43 is recruited to the same loci as SIRT2, however, as we observe global cellular effects such as H3K18 deacetylation by immunoblot and changes to cell viability, we predict that other loci in addition to the subset of TSSs we previously identified are also likely to be targeted. In addition to TSSs, H3K18 acetylation has also been linked to the regulation of non-gene chromosomal elements which might not be detected by examining transcriptional changes of protein-coding genes. These include LINE and Alu retrotransposons [56,57], pericentric satellite DNA [11] and it has been proposed to regulate rDNA [58]. Similarly, R-loops can preferentially form at similar genomic sites such as rDNA, tRNA, transposable elements, GAC triple repeats and satellite DNA repeat elements [48]. These additional features across the genome therefore could also be potential targets of SIRT2, which might contribute to the global effects observed during infection.

Recently SIRT2 has been shown to induce H3K18 deacetylation in macrophages infected with *M*. *tuberculosis* [59]. In this context SIRT2 also functions to promote bacterial infection and likewise its chemical inhibition causes a reduction in bacterial burden [59]. Considering these findings, SIRT2, and now TDP-43, could represent potential targets for host directed therapeutics to promote bacterial clearance. In the context of *L*. *monocytogenes* infection treatment with a SIRT2 inhibitor would preferentially induce the death of infected cells which could then be more effectively cleared by immune cells or used in combination with traditional antibacterial therapeutics. Indeed, our results show that over short time frames (24 hours) inhibition of SIRT2 does not appear to influence uninfected cells and is only detrimental to those with a high bacterial burden.

Beyond infection these factors are also independently linked to many other human pathologies, and mutations in SIRT2, TDP-43 and R-loop regulating factors have been linked with age-related illnesses such as cancer and neurodegenerative diseases, both of which are also intrinsically linked to the deregulation of DNA damage responses. As such, these mechanisms not only have implications in better understanding cellular response to infection but could also extend to other factors of human health and disease.

## Materials and methods

### Ethics statement

Protocols for animal studies were reviewed and approved by the Comité d’Ethique pour l’Expérimentation Animale of Institut Pasteur under approval number Dap170005 and performed in accordance with national laws and institutional guidelines for animal care and use.

### Cell Culture, inhibitor treatments and Listeria monocytogenes infections

HeLa (ATCC, CCL-2) cells were grown to semi-confluency in minimum essential medium (MEM) plus GlutaMAX (Gibco) supplemented with 1 mM sodium pyruvate (Gibco), and 10% fetal bovine serum (FBS). 24 hours before infection, HeLa cell medium was changed to low serum (0.25% FBS) MEM medium containing 1 mM sodium pyruvate. MEFs were grown to confluency in Dulbecco’s Modified Eagle Medium (DMEM) plus GlutaMAX (Gibco) supplemented with 1 mM sodium pyruvate (Gibco), and 10% fetal bovine serum (FBS). Infection of MEFs was carried out in low serum DMEM for 1 hour, cells were then washed and incubated in complete medium containing 10 μg.mL^-1^. *Listeria monocytogenes* strains (see S1 Table) were grown overnight in brain heart infusion (BHI) liquid broth with shaking at 37°C. For infection, bacteria were subcultured (1 in 10) into fresh BHI and grown to mid log phase (OD_600_ = 0.8–1) and washed 3× in MEM + 0.25% FBS before being added to cells. Bacteria were then added at a MOI of 100 for HeLa cells and 50 for MEFs then incubated for 1 hour. Cells were then washed 3× in MEM + 0.25% FBS and incubated in fresh medium for 30 minutes prior to the addition of 10 μg.mL^-1^ gentamicin for the remaining time of the infection. Inhibitors were added 2 hours prior to infection and remained present until 1 hour post-infection when cells were washed. SIRT2 inhibitor AGK2 (Calbiochem) was used at a concentration of 5 mM.

### Cell viability alamarBlue assay

Cells were incubated at 37°C in fresh medium containing 10% alamarBlue reagent for 1–2 hours. Fluorescence (Ex/Em 560/590 nm) was then read using a Cytation 5 (BioTek). Fluorescence readings were blank corrected to wells containing only culture medium and results are expressed as a percentage of uninfected cell viability.

### Immunofluorescence microscopy

For immunofluorescence HeLa cells were plated onto coverslips prior to treatments. Following treatments, cells were washed three times in PBS and fixed using 4% PFA in DPBD for 10 mins. Cells were then permeabilised for 10 mins in 0.2% Trition X-100 PBS. Coverslips were then incubated in blocking buffer (1% BSA TBS) for 1 hour. For immunostaining, coverslips were inverted on to droplets of blocking buffer containing Phospho-Histone H2A.X (Ser139) (CST, 2577) antibody (1:500) then incubated in a humidified chamber overnight at 4°C. Subsequently, coverslips were washed 3 times in PBS + 0.1% Tween then incubated at room temperature in the dark for 1 hour in blocking buffer containing Alexa Fluor 546 goat anti-rabbit IgG (Invitrogen, A-11035) secondary antibody (1:1500) for 1 hour. Coverslips were washed three times TBS + 0.1% Tween, nuclei were stained with 300 nM (100 ng.mL^-1^) Hoechst 33342 for 15 mins. Coverslips were then washed three times in TBS, rinsed briefly in distilled water and mounted using Fluoromount-G Mounting Medium (INTERCHIM). All images were acquired using a Zeiss Axio Observer spinning-disk confocal microscope driven by the MetaMorph software. For quantification a minimum of ten fields of view were obtained per condition of each biological replicate.

### Immunoblotting and band quantification

Cell lysates were prepared in 2× Laemmli loading buffer supplemented with cOmplete protease inhibitor and PhosSTOP phosphatase inhibitor tablets (Roche), 1 mM PMSF, 5 mM sodium butyrate and 5% β -mercaptoethanol. Proteins were separated by SDS-PAGE using TrisGlycine buffer systems and transferred to PVDF membranes (Bio-Rad Laboratories). Membranes were blocked for 1 hour in TBS + 0.1% Tween containing 5% milk and then incubated with primary antibodies (as per the manufacturer’s instructions) overnight at 4°C with rocking. Immunoblot quantification used images acquired on a Chemidoc MP (Bio-Rad), analyzed using Image Lab software (Bio-Rad Laboratories).

### Antibodies

Antibodies used in this study are as follows; anti-GFP antibody (Abcam, ab290), Acetyl-Histone H3 (Lys18) antibody (CST, 9675), anti-Histone H3 antibody (Abacam, ab1791), anti-β-actin (Sigma, AC-15), anti-TDP-43 antibody (Sigma, T1705), anti-SIRT2 (CST, 12650) anti-mCherry antibody (1C51) (Novus Biologicals, NBP1-96752), anti-γH2A.X (S139) antibody (2OE3) (CST, 9718S), anti-H2A.X antibody (CST, 2595S), Phospho-Histone H2A.X (Ser139) antibody (immunofluorescence) (CST, 2577).

### *In vivo* animal studies

Wild-type C57BL/6 mice were purchased from Janvier Labs. *Sirt2*^tm1a(EUCOMM)Wtsi^ mice were obtained from the Sanger Center. For details, see www.informatics.jax.org/javawi2/servlet/WIFetch?page=alleleDetail&key=606707. Female mice aged 8–16 weeks old were infected by intravenous injection of 10^5^ bacteria per animal which proceeded for 72 hours.

### Cell cycle analysis with PI by FACS

For cell cycle analysis, cells were collected using PBS + 5 mM EDTA and washed once in PBS. Cells were then fixed in cold 70% ethanol for 30 minutes at 4°C. Fixed cells were washed twice in PBS then treated with 100 μg/mL of RNaseA for 30 min at room temperature. DNA was then stained for 30 minutes using 10 μg/mL Propidium Iodide and cells. Flow cytometry acquisitions were performed on a BD LSRFortessa Cell Analyzer. Data were analysed using FlowJo software.

### RNA interference and DNA transfections

Transient RNAi was carried out using ON-TARGETplus siRNAs from Dharmacon. HeLa cells were transfected with siRNA targeting either *SIRT2* (SMARTpool L-004826-00-0005), or *TARDBP* (SMARTpool L-012394-00-0005). ON-TARGETplus Non-targeting Pool siRNA (D-001810-10-05) served as the negative control. Reverse transfections were performed in 6 well plates using Lipofectamine RNAiMAX reagent (Invitrogen). Briefly, 2.5x10^5^ HeLa were added to wells containing 15 pmol of siRNA mixed with 3 μL Lipofectamine RNAiMAX in 500 μL OptiMEM (Gibco) and incubated for 48 hours prior to further treatment or infection.

Transient expression of DNA plasmids was carried out in 6 well plates by reverse transfection using Lipofectamine LTX (Invitrogen). Briefly, 5-6x10^5^ HeLa cells were added to wells containing DNA-lipid complexes consisting of 1 μg plasmid DNA mixed with 1.5 μL Plus reagent and 3 μL LTX transfection reagent in 500 μL OptiMEM.

### RNaseH1 transfection and induction

For experiments testing the role RNaseH1 overexpression HeLa stably expressing the tetracycline repressor (HeLa T-Rex) protein (Agathe Subtil) were used to enable induction of pICE plasmids. HeLa T-Rex cells were transfected as described above. For plasmid induction, transfected cells were incubated overnight with 10 ng.mL^-1^ Anhydrotetracycline hydrochloride (AHT).

### Co-immunoprecipitation with MNase lysis

Immunoprecipitations of SIRT2-GFP were performed using GFP-Trap agarose beads (Chromotek). HeLa cells transfected with tagged-SIRT2 or empty pEGFP-N1 or HeLa cells stably expressing SIRT2-GFP and transfected with pICE-NLS-mCherry or pICE-RNaseHI-WT-NLS-mCherry were used. 24 hours post-transfection cells were infected as described above for 3 hours. Cells were then collected by scraping in PBS and resuspended in 100uL MNase reaction buffer (1mM CaCl_2_, 0.2% NP-40, 50mM Tris-HCl (pH 7.6) 1mM CaCl_2_, 0.2% NP-40, 50mM Tris-HCl (pH 7.6). Cell suspensions were agitated by pipetting and nuclei were collected by “pop-spin” [60]. Isolated nuclei were then resuspended in fresh MNase reaction buffer containing 10 U micrococcal nuclease and incubated at 37 °C for 20 min. Reaction was terminated with 5 mM EDTA and samples were diluted 1:1 with 2X RIPA buffer containing 1 mM PMSF and sodium butyrate and incubated on ice for 10 minutes. Lysate was cleared by centrifugation at 20000 *xg* for 5 minutes and the resulting supernatant was diluted with 600 μL of wash/dilution buffer (10 mM Tris/Cl pH 7.5; 150 mM NaCl; 0.5 mM EDTA). 40 μL was removed for input and the remaining lysate was incubated with GFP-Trap agarose beads at 4°C with agitation for 1 hour. The beads were washed twice in wash buffer and once in wash buffer containing 300 mM NaCl. Proteins were eluted by boiling beads in 50 μL 2× Laemmli buffer with 5% β-mercaptoethanol.

### Chromatin immunoprecipitation PCR

3-5x10^6^ cells were cross-linked at room temperature with 1% formaldehyde for 10 minutes followed by quenching with 130 mM glycine for 5 minutes. Chromatin extraction and ChIP-PCR were performed as previously described with slight modifications [61] for buffer details see S2 Table. Briefly, cell pellets were lysed on ice in nuclear isolation buffer (NIB) supplemented with 0.2% Triton X-100 and inhibitors (1× cOmplete protease, 1X PhosSTOP, 10 mM sodium butyrate, 0.2mM PMSF) for 30 min with gentle pipetting every 10 min. Nuclei were collected by centrifugation and re-suspended in chromatin shearing buffer with inhibitors. Chromatin was fragmented by sonication (30 cycles of 15 s ‘on’ and 30 s ‘off’) with a Bioruptor (Diagenode) to 200–1000 bp. Sheared chromatin was cleared by centrifugation, sampled for size using 2% agarose gel electrophoresis and quantified using Pico488 (Lumiprobe, 42010). 2 μg of antibody (anti-TDP-43, T1705; anti-GFP antibody, ab290) was used per ChIP and were bound to Dynabeads Protein G (Invitrogen) overnight at 4°C with gentle rotation. Chromatin was diluted to 10–15 μg/IP with SDS dilution buffer supplemented with inhibitors. 8% of ChIP sample volume was reserved to serve as input. Diluted chromatin was then added to antibody bound Dynabeads and incubated at 4°C overnight with gentle rotation. IP samples were washed sequentially with 1 mL of buffers 1–6. Water containing 10% Chelex was added to washed beads and input samples and were eluted and de-crosslinked by boiling for 10 minutes. Samples were then treated with RNase A at room temperature for 10 minutes at 37°C followed by proteinase K (500 μg/ml) for 20 min at 55°C. Samples were then boiled for a further 10 min and recovered DNA was purified by phenol–chloroform extraction and isopropanol precipitation and resuspended in molecular grade water. ChIP DNA was quantified by qRT-PCR using iTaq Universal SYBR Green Supermix and results were expressed as percent recovery from input calculated as 2 raised to cycle adjusted input sample quantitation cycle (Cq) value minus the Cq immunoprecipitation sample, multiplied by 100. For buffer formulations and primer sequences see S2 and S3 Tables respectively.

### RNA isolation, reverse transcription, and qRT-PCR

RNA was extracted from cells using TRIzol Reagent (Life Technologies) extraction method as per the manufacturer’s instructions. cDNA was synthesised from 2 μg purified RNA using iScript cDNA Synthesis Kit (Bio-Rad) and quantified by qRT-PCR using iTaq Universal SYBR Green Supermix. Data was analysed using ΔCT method relative to *GAPDH*.

### Plasmids single, oligo mutagenesis and molecular cloning

Routine cloning was carried out by sequence- and ligation-independent cloning (SLIC) [62] for primers see S3 Table. For further details on plasmids used in this study see S4 Table. pEGFP-H3 WT, pEGFP-H3 K18A, pEGFP-H3 K18Q were a gift from Dr Fang-Lin Sun [63]. pmCherry TDP-43 was cloned from TDP43 NOTAG1(Addgene #28206) which was a gift from Zuoshang Xu [64]. pICE-NLS-mCherry (Addgene #60364) and pICE-RNaseHI-WT-NLS-mCherry (Addgene #60365) were gifts from Patrick Calsou [65]. pICE-RNaseHI-WT-NLS-mCherry (Dead) was made by introducing inactivating D10R and E48R mutations by single oligo mutagenesis [66].

### Statistical analysis

All experiments were repeated at least twice, and statistical tests are reported in the figure legends. Data normality was tested by Shapiro-Wilk test, and appropriate parametric or non-parametric tests were used. Data plots and statistics were generated using Prism (version 9, GraphPad Software Inc.).

## Supporting information

S1 Fig SIRT2 promotes host cell viability and *Listeria* infection. **(A)** Enumeration of live (Trypan negative) and dead (Trypan positive) HeLa cells pre-treated for 2 hours with DMSO or 5 mM AGK2 and infected for stated times. Cells were enumerated with Countess II Automated Cell Counter from 2 independent experiments. Statistical significance of live cells was determined by two-way ANOVA with FDR Benjamini-Hochberg (BH) correction for multiple comparisons (ns = not significant, * = *p* < 0.05, ** = *p* < 0.001), *** = *p* < <0.0001). **(B)** Quantification of *L*. *monocytogenes* intracellular CFUs from HeLa cells infected for 2.5 h or 24 h. Data are presented as CFU/well. Graphs display mean CFU ± SEM from 2 independent experiments. Statistical significance was determined by one-way ANOVA with FDR Benjamini-Hochberg (BH) correction for multiple comparisons (ns = not significant, *** = *p* < 0.001). **(C)** Quantification of *L*. *monocytogenes* intracellular CFUs from MEFs infected for 2.5 h, 6 h or 24 h. Data are presented as CFU/well. Graphs display mean CFU ± SEM from 2 independent experiments. Statistical significance was determined by one-way ANOVA with FDR Benjamini-Hochberg (BH) correction for multiple comparisons (ns = not significant, * = *p* < <0.05). **(D)** Representative images from S1 Movie.
(TIF)

S2 Fig SIRT2 inhibition affects DNA damage accumulation during infection. **(A)** Percentage of γH2Ax positive cells from Fig 1B. Error bars represent the SEM from four independent experiments. Statistical significance was calculated by two-way ANOVA with FDR Benjamini-Hochberg (BH) correction for multiple comparisons (ns = not significant, * = *p* < 0.05). **(B)** Immunoblot detection of γH2Ax and total H2Ax (left) from whole cell lysates of HeLa cells left uninfected (-) or infected with increasing MOIs of *L*. *monocytogenes* (*Lm MOI*) for 24 hours. (Above) Quantification of γH2Ax levels relative to uninfected. **(C)** Immunoblot detection of γH2Ax and total H2Ax (above) from whole cell lysates of wildtype and *Sirt2*^*-/*-^ MEF cells left uninfected (-) or infected with increasing *L*. *monocytogenes* for stated timepoints. (Below) Quantification of γH2Ax levels relative to uninfected. Graph shows the mean ± SEM from at least three independent experiments. Statistical significance was determined by one-way ANOVA with FDR Benjamini-Hochberg (BH) correction for multiple comparisons (ns = not significant, ** = *p* < <0.005, *** = *p* < <0.001), **** = *p* < <0.0001.
(TIF)

S3 Fig SIRT2 inhibition and infection does not affect cell cycle. FACS analysis of propidium iodide-stained cells HeLa cells. Cells are untreated or treated with AGK2 then left uninfected or infected for stated times. Percentage of cells in each stage of the cell cycle is calculated with Flowjo software and presented with each histogram as % G0/G1, % S and % G2/M. Representative histograms of two independent experiments.
(TIF)

S4 Fig SIRT2 and H3K18 deacetylation protect infected cells from infection induced DNA damage. **(A)** Immunoblot detection of stated proteins from uninfected mouse spleen lysates (left). Quantification of normalised H3K18-ac and γH2Ax levels. Graphs show collated values from 4 mice, box and whisker plot with solid line denoting the median value. Statistical significance was determined by Two-tailed Unpaired t test (* = *p* < 0.05, *** = *p* <0.001). **(B)** Total *L*. *monocytogenes* CFU per spleen extracted from *wildtype* and *Sirt2*^*-/-*^ mice 72 hours post infection. **(C)** Immunoblot detection of InlC (left) from whole cell lysates of HeLa cells left uninfected (-) or infected with *L*. *monocytogenes (Lm)* for 24 hours. Cells are expressing stated H3-GFP plasmids and treated with DMSO or 5 mM AGK2. Quantification of InlC levels (right) relative to uninfected. Results are expressed as intensity of actin normalised InlC. Graph shows the mean ± SEM from three independent experiments. Statistical significance was determined by two-way with FDR Benjamini-Hochberg (BH) correction for multiple comparisons (ns = not significant, * = *p* <0.05).
(TIF)

S5 Fig Identification of SIRT2-interacting partners shown to localise to SIRT2-regulated genes by ChIP-seq. Venn diagrams illustrating proteins shared between SIRT2-interactome and interactors of the TSSs of *MYLIP*, *ERRC5*, *LEF1*, *SYDE2*, *EHHADH* and *ARAP2*.
(TIF)

S6 Fig Phosphorylation of SIRT2 at S25 modulates interactions with TDP-43. HeLa cells expressing either mCherry alone or mCherry-TDP-43 were co-transfected with stated variants of SIRT2-GFP followed by immunoprecipitation using RFP-Trap agarose beads. Cell lysates (Input) and IP fractions were immunoblotted using antibodies against TDP-43 or GFP for detection of SIRT2.
(TIF)

S7 Fig Knockdown of SIRT2 or TDP-43 reduces long term efficacy of *Listeria* infection. **(A)** Western blot showing TDP-43 knockdown following siRNA transfection for 48 hours. Relative mRNA expression of **(B)** *SIRT2* and **(C)** *TARDBP* as detected by qPCR normalised to GAPDH. Mean ± S.E.M from three independent experiments are plotted. **(D)** Quantification of *L*. *monocytogenes* intracellular CFUs. HeLa cells were transfected with indicated siRNAs and infected for 2.5 h or 24 h. Lysates were plated onto BHI agar and bacterial CFUs were enumerated. Data are presented as CFU/well. Individual biological replicates are plotted as paired values.
(TIF)

S8 Fig Blocking R-loop formation reduces long term efficacy of *Listeria* infection. Quantification of *L*. *monocytogenes* intracellular CFU/cell. HeLa cells expressing either mCherry or RNaseH1 were treated with DMSO or 5 mM AGK2 then infected with *L*. *monocytogenes*. Intracellular bacteria were extracted at 2.5, 6 and 24 hours post infection plated onto BHI agar and bacterial CFUs were enumerated. Data are presented as average CFU/cell. Mean ± S.E.M from three independent experiments are plotted.
(TIF)

S1 Movie SIRT2 promotes host cell viability and *Listeria* infection. Wildtype and *Sirt2*^*-/-*^ MEFs were infected with *L*. *monocytogenes* expressing GFP and imaged once per hour between 6–24 hours post infection. Images were acquired using a Cytation 5 Cell Imaging Multi-Mode Reader.
(MOV)

S1 Table Bacterial strains used in study. (TIF)

S2 Table Recipes of buffers used in this study. (TIF)

S3 Table Primers used in this study. (TIF)

S4 Table Plasmids used in this study. (TIF)

## References

[1] Houtkooper RH, Pirinen E, Auwerx J. Sirtuins as regulators of metabolism and healthspan. Nat Rev Mol Cell Biol. 2012;13: 225–238. doi: 10.1038/nrm3293 22395773 PMC4872805

[2] Gomes P, Fleming Outeiro T, Cavadas C. Emerging Role of Sirtuin 2 in the Regulation of Mammalian Metabolism. Trends Pharmacol Sci. 2015;36: 756–768. doi: 10.1016/j.tips.2015.08.001 26538315

[3] Eskandarian HA, Impens F, Nahori M-A, Soubigou G, Coppée J-Y, Cossart P, et al. A Role for SIRT2-Dependent Histone H3K18 Deacetylation in Bacterial Infection. Science (80-). 2013;341: 1238858. doi: 10.1126/science.1238858 23908241

[4] Pereira JM, Chevalier C, Chaze T, Gianetto Q, Impens F, Matondo M, et al. Infection Reveals a Modification of SIRT2 Critical for Chromatin Association. Cell Rep. 2018;23: 1124–1137. doi: 10.1016/j.celrep.2018.03.116 29694890 PMC5946459

[5] Eldridge MJG, Pereira JM, Impens F, Hamon MA. Active nuclear import of the deacetylase Sirtuin-2 is controlled by its C-terminus and importins. Sci Rep. 2020;10: 1–12. doi: 10.1038/s41598-019-56847-4 32042025 PMC7010746

[6] Wang RH, Sengupta K, Li C, Kim HS, Cao L, Xiao C, et al. Impaired DNA Damage Response, Genome Instability, and Tumorigenesis in SIRT1 Mutant Mice. Cancer Cell. 2008;14: 312–323. doi: 10.1016/j.ccr.2008.09.001 18835033 PMC2643030

[7] Jeong J, Juhn K, Lee H, Kim SH, Min BH, Lee KM, et al. SIRT1 promotes DNA repair activity and deacetylation of Ku70. Exp Mol Med. 2007;39: 8–13. doi: 10.1038/emm.2007.2 17334224

[8] Chen L, Huang S, Lee L, Davalos A, Schiestl RH, Campisi J, et al. WRN, the protein deficient in Werner syndrome, plays a critical structural role in optimizing DNA repair. Aging Cell. 2003;2: 191–199. doi: 10.1046/j.1474-9728.2003.00052.x 12934712

[9] Fan W, Luo J. SIRT1 regulates UV-induced DNA repair through deacetylating XPA. Mol Cell. 2010;39: 247–258. doi: 10.1016/j.molcel.2010.07.006 20670893

[10] Onn L, Portillo M, Ilic S, Cleitman G, Stein D, Kaluski S, et al. SIRT6 is a DNA double-strand break sensor. Elife. 2020;9. doi: 10.7554/eLife.51636 31995034 PMC7051178

[11] Tasselli L, Xi Y, Zheng W, Tennen RI, Odrowaz Z, Simeoni F, et al. SIRT6 deacetylates H3K18ac at pericentric chromatin to prevent mitotic errors and cellular senescence. Nat Struct Mol Biol. 2016;23: 434–440. doi: 10.1038/nsmb.3202 27043296 PMC5826646

[12] Vazquez BN, Thackray JK, Simonet NG, Kane-Goldsmith N, Martinez-Redondo P, Nguyen T, et al. SIRT 7 promotes genome integrity and modulates non-homologous end joining DNA repair. EMBO J. 2016;35: 1488–1503. doi: 10.15252/embj.201593499 27225932 PMC4884211

[13] Kim HS, Patel K, Muldoon-Jacobs K, Bisht KS, Aykin-Burns N, Pennington JD, et al. SIRT3 Is a Mitochondria-Localized Tumor Suppressor Required for Maintenance of Mitochondrial Integrity and Metabolism during Stress. Cancer Cell. 2010;17: 41–52. doi: 10.1016/j.ccr.2009.11.023 20129246 PMC3711519

[14] Cheng Y, Ren X, Gowda ASP, Shan Y, Zhang L, Yuan YS, et al. Interaction of Sirt3 with OGG1 contributes to repair of mitochondrial DNA and protects from apoptotic cell death under oxidative stress. Cell Death Dis. 2013;4: e731–e731. doi: 10.1038/cddis.2013.254 23868064 PMC3730425

[15] Jeong SM, Xiao C, Finley LWS, Lahusen T, Souza AL, Pierce K, et al. SIRT4 has tumor-suppressive activity and regulates the cellular metabolic response to dna damage by inhibiting mitochondrial glutamine metabolism. Cancer Cell. 2013;23: 450–463. doi: 10.1016/j.ccr.2013.02.024 23562301 PMC3650305

[16] Lemos V, de Oliveira RM, Naia L, Szegö É, Ramos E, Pinho S, et al. The NAD+-dependent deacetylase SIRT2 attenuates oxidative stress and mitochondrial dysfunction and improves insulin sensitivity in hepatocytes. Hum Mol Genet. 2017;26: 4105–4117. doi: 10.1093/hmg/ddx298 28973648

[17] de Oliveira RM, Sarkander J, Kazantsev AG, Outeiro TF. SIRT2 as a Therapeutic Target for Age-Related Disorders. Front Pharmacol. 2012;3: 82. doi: 10.3389/fphar.2012.00082 22563317 PMC3342661

[18] Serrano L, Martínez-Redondo P, Marazuela-Duque A, Vazquez BN, Dooley SJ, Voigt P, et al. The tumor suppressor SirT2 regulates cell cycle progression and genome stability by modulating the mitotic deposition of H4K20 methylation. Genes Dev. 2013;27: 639–653. doi: 10.1101/gad.211342.112 23468428 PMC3613611

[19] Kim HS, Vassilopoulos A, Wang RH, Lahusen T, Xiao Z, Xu X, et al. SIRT2 Maintains Genome Integrity and Suppresses Tumorigenesis through Regulating APC/C Activity. Cancer Cell. 2011;20: 487–499. doi: 10.1016/j.ccr.2011.09.004 22014574 PMC3199577

[20] Zhang H, Park SH, Pantazides BG, Karpiuk O, Warren MD, Hardy CW, et al. SIRT2 directs the replication stress response through CDK9 deacetylation. Proc Natl Acad Sci U S A. 2013;110: 13546–13551. doi: 10.1073/pnas.1301463110 23898190 PMC3746840

[21] Leitão E, Costa AC, Brito C, Costa L, Pombinho R, Cabanes D, et al. Listeria monocytogenes induces host DNA damage and delays the host cell cycle to promote infection. Cell Cycle. 2014;13: 928–940. doi: 10.4161/cc.27780 24552813 PMC3984316

[22] Samba-Louaka A, Pereira JM, Nahori MA, Villiers V, Deriano L, Hamon MA, et al. Listeria monocytogenes Dampens the DNA Damage Response. PLoS Pathog. 2014;10: 1004470. doi: 10.1371/journal.ppat.1004470 25340842 PMC4207825

[23] Ashida H, Mimuro H, Ogawa M, Kobayashi T, Sanada T, Kim M, et al. Cell death and infection: A double-edged sword for host and pathogen survival. J Cell Biol. 2011;195: 931–942. doi: 10.1083/jcb.201108081 22123830 PMC3241725

[24] Friedrich A, Pechstein J, Berens C, Lührmann A. Modulation of host cell apoptotic pathways by intracellular pathogens. Curr Opin Microbiol. 2017;35: 88–99. doi: 10.1016/j.mib.2017.03.001 28319728

[25] Pirbhai M, Dong F, Zhong Y, Pan KZ, Zhong G. The Secreted Protease Factor CPAF Is Responsible for Degrading Pro-apoptotic BH3-only Proteins in Chlamydia trachomatis-infected Cells. J Biol Chem. 2006;281: 31495–31501. doi: 10.1074/jbc.M602796200 16940052

[26] Behar SM, Briken V. Apoptosis inhibition by intracellular bacteria and its consequence on host immunity. Curr Opin Immunol. 2019;60: 103–110. doi: 10.1016/j.coi.2019.05.007 31228759 PMC6800630

[27] Knodler LA, Finlay B, Steele-Mortimer O. The Salmonella effector protein SopB protects epithelial cells from apoptosis by sustained activation of Akt. J Biol Chem. 2005;280: 9058–9064. doi: 10.1074/jbc.M412588200 15642738

[28] Yan F, Cao H, Chaturvedi R, Krishna U, Hobbs SS, Dempsey PJ, et al. Epidermal Growth Factor Receptor Activation Protects Gastric Epithelial Cells From Helicobacter pylori-Induced Apoptosis. Gastroenterology. 2009;136: 1297–1307.e3. doi: 10.1053/j.gastro.2008.12.059 19250983 PMC2878739

[29] Kühbacher A, Gouin E, Mercer J, Emmenlauer M, Dehio C, Cossart P, et al. Imaging InlC Secretion to Investigate Cellular Infection by the Bacterial Pathogen Listeria monocytogenes. JoVE (Journal Vis Exp. 2013; e51043. doi: 10.3791/51043 24084755 PMC3923893

[30] Davis CA, Hitz BC, Sloan CA, Chan ET, Davidson JM, Gabdank I, et al. The Encyclopedia of DNA elements (ENCODE): data portal update. Nucleic Acids Res. 2018;46: D794–D801. doi: 10.1093/nar/gkx1081 29126249 PMC5753278

[31] Morales AJ, Carrero JA, Hung PJ, Tubbs AT, Andrews JM, Edelson BT, et al. A type I IFN-dependent DNA damage response regulates the genetic program and inflammasome activation in macrophages. Elife. 2017;6. doi: 10.7554/eLife.24655 28362262 PMC5409825

[32] Kitamura A, Shibasaki A, Takeda K, Suno R, Kinjo M. Analysis of the substrate recognition state of TDP-43 to single-stranded DNA using fluorescence correlation spectroscopy. Biochem Biophys Reports. 2018;14: 58–63. doi: 10.1016/j.bbrep.2018.03.009 29872735 PMC5986658

[33] Kuo PH, Chiang CH, Wang YT, Doudeva LG, Yuan HS. The crystal structure of TDP-43 RRM1-DNA complex reveals the specific recognition for UG- and TG-rich nucleic acids. Nucleic Acids Res. 2014;42: 4712–4722. doi: 10.1093/nar/gkt1407 24464995 PMC3985631

[34] Buratti E, Brindisi A, Pagani F, Baralle FE. Nuclear factor TDP-43 binds to the polymorphic TG repeats in CFTR intron 8 and causes skipping of exon 9: A functional link with disease penetrance. Am J Hum Genet. 2004;74: 1322–1325. doi: 10.1086/420978 15195661 PMC1182100

[35] Buratti E, Baralle FE. Characterization and Functional Implications of the RNA Binding Properties of Nuclear Factor TDP-43, a Novel Splicing Regulator of CFTR Exon 9. J Biol Chem. 2001;276: 36337–36343. doi: 10.1074/jbc.M104236200 11470789

[36] Buratti E, Dörk T, Zuccato E, Pagani F, Romano M, Baralle FE. Nuclear factor TDP-43 and SR proteins promote in vitro and in vivo CFTR exon 9 skipping. EMBO J. 2001;20: 1774–1784. doi: 10.1093/emboj/20.7.1774 11285240 PMC145463

[37] Alami NH, Smith RB, Carrasco MA, Williams LA, Winborn CS, Han SSW, et al. Axonal Transport of TDP-43 mRNA Granules Is Impaired by ALS-Causing Mutations. Neuron. 2014;81: 536–543. doi: 10.1016/j.neuron.2013.12.018 24507191 PMC3939050

[38] Lagier-Tourenne C, Polymenidou M, Cleveland DW. TDP-43 and FUS/TLS: Emerging roles in RNA processing and neurodegeneration. Hum Mol Genet. 2010;19: 46–64. doi: 10.1093/hmg/ddq137 20400460 PMC3167692

[39] Ou SH, Wu F, Harrich D, García-Martínez LF, Gaynor RB. Cloning and characterization of a novel cellular protein, TDP-43, that binds to human immunodeficiency virus type 1 TAR DNA sequence motifs. J Virol. 1995;69: 3584–3596. doi: 10.1128/JVI.69.6.3584-3596.1995 7745706 PMC189073

[40] Lalmansingh AS, Urekar CJ, Reddi PP. TDP-43 is a transcriptional repressor: The testis-specific mouse acrv1 gene is a TDP-43 target in vivo. J Biol Chem. 2011;286: 10970–10982. doi: 10.1074/jbc.M110.166587 21252238 PMC3064152

[41] Chang ke C, Wu TH, Wu CY, Chiang hui M, Toh EKW, Hsu YC, et al. The N-terminus of TDP-43 promotes its oligomerization and enhances DNA binding affinity. Biochem Biophys Res Commun. 2012;425: 219–224. doi: 10.1016/j.bbrc.2012.07.071 22835933

[42] Afroz T, Hock E-M, Ernst P, Foglieni C, Jambeau M, Gilhespy LAB, et al. Functional and dynamic polymerization of the ALS-linked protein TDP-43 antagonizes its pathologic aggregation. Nat Commun 2017 81. 2017;8: 1–15. doi: 10.1038/s41467-017-00062-0 28663553 PMC5491494

[43] Jo M, Lee S, Jeon YM, Kim S, Kwon Y, Kim HJ. The role of TDP-43 propagation in neurodegenerative diseases: integrating insights from clinical and experimental studies. Exp Mol Med. 2020;52: 1652–1662. doi: 10.1038/s12276-020-00513-7 33051572 PMC8080625

[44] Mitra J, Guerrero EN, Hegde PM, Liachko NF, Wang H, Vasquez V, et al. Motor neuron disease-associated loss of nuclear TDP-43 is linked to DNA double-strand break repair defects. Proc Natl Acad Sci U S A. 2019;116: 4696–4705. doi: 10.1073/pnas.1818415116 30770445 PMC6410842

[45] Konopka A, Whelan DR, Jamali MS, Perri E, Shahheydari H, Toth RP, et al. Impaired NHEJ repair in amyotrophic lateral sclerosis is associated with TDP-43 mutations. Mol Neurodegener. 2020;15: 1–28. doi: 10.1186/s13024-019-0350-4 32907630 PMC7488163

[46] Marnef A, Legube G. R-loops as Janus-faced modulators of DNA repair. Nat Cell Biol. 2021;23: 305–313. doi: 10.1038/s41556-021-00663-4 33837288

[47] Crossley MP, Bocek M, Cimprich KA. R-Loops as Cellular Regulators and Genomic Threats. Mol Cell. 2019;73: 398–411. doi: 10.1016/j.molcel.2019.01.024 30735654 PMC6402819

[48] Niehrs C, Luke B. Regulatory R-loops as facilitators of gene expression and genome stability. Nat Rev Mol Cell Biol. 2020. doi: 10.1038/s41580-019-0206-3 32005969 PMC7116639

[49] Gan W, Guan Z, Liu J, Gui T, Shen K, Manley JL, et al. R-loop-mediated genomic instability is caused by impairment of replication fork progression. Genes Dev. 2011;25: 2041–2056. doi: 10.1101/gad.17010011 21979917 PMC3197203

[50] Cristini A, Ricci G, Britton S, Salimbeni S, Huang S yin N, Marinello J, et al. Dual Processing of R-Loops and Topoisomerase I Induces Transcription-Dependent DNA Double-Strand Breaks. Cell Rep. 2019;28: 3167–3181.e6. doi: 10.1016/j.celrep.2019.08.041 31533039 PMC8274950

[51] Chakraborty A, Tapryal N, Venkova T, Horikoshi N, Pandita RK, Sarker AH, et al. Classical non-homologous end-joining pathway utilizes nascent RNA for error-free double-strand break repair of transcribed genes. Nat Commun. 2016;7: 1–12. doi: 10.1038/ncomms13049 27703167 PMC5059474

[52] Yasuhara T, Kato R, Hagiwara Y, Shiotani B, Yamauchi M, Nakada S, et al. Human Rad52 Promotes XPG-Mediated R-loop Processing to Initiate Transcription-Associated Homologous Recombination Repair. Cell. 2018;175: 558–570.e11. doi: 10.1016/j.cell.2018.08.056 30245011

[53] Gianini M, Bayona-Feliu A, Sproviero D, Barroso SI, Cereda C, Aguilera A. TDP-43 mutations link Amyotrophic Lateral Sclerosis with R-loop homeostasis and R loopmediated DNA damage. PLoS Genet. 2020;16: e1009260. doi: 10.1371/journal.pgen.1009260 33301444 PMC7755276

[54] Mosler T, Conte F, Mikicic I, Kreim N, Möckel MM, Flach J, et al. R-loop proximity proteomics identifies a role of DDX41 in transcription-1 associated genomic instability. 2021 Apr. doi: 10.21203/RS.3.RS-337351/V1 PMC8677849 34916496

[55] Cristini A, Groh M, Kristiansen MS, Gromak N. RNA/DNA Hybrid Interactome Identifies DXH9 as a Molecular Player in Transcriptional Termination and R-Loop-Associated DNA Damage. Cell Rep. 2018;23: 1891–1905. doi: 10.1016/j.celrep.2018.04.025 29742442 PMC5976580

[56] Vazquez BN, Thackray JK, Simonet NG, Chahar S, Kane-Goldsmith N, Newkirk SJ, et al. SIRT7 mediates L1 elements transcriptional repression and their association with the nuclear lamina. Nucleic Acids Res. 2019;47: 7870. doi: 10.1093/nar/gkz519 31226208 PMC6735864

[57] Ferrari R, de Llobet Cucalon LI, Di Vona C, Le Dilly F, Vidal E, Lioutas A, et al. TFIIIC Binding to Alu Elements Controls Gene Expression via Chromatin Looping and Histone Acetylation. Mol Cell. 2020;77: 475–487.e11. doi: 10.1016/j.molcel.2019.10.020 31759822 PMC7014570

[58] Paredes S, Angulo-Ibanez M, Tasselli L, Carlson SM, Zheng W, Li TM, et al. The epigenetic regulator SIRT7 guards against mammalian cellular senescence induced by ribosomal DNA instability. J Biol Chem. 2018;293: 11242–11250. doi: 10.1074/jbc.AC118.003325 29728458 PMC6052228

[59] Bhaskar A, Kumar S, Khan MZ, Singh A, Dwivedi VP, Nandicoori VK. Host sirtuin 2 as an immunotherapeutic target against tuberculosis. Elife. 2020;9: 1–28. doi: 10.7554/eLife.55415 32697192 PMC7398663

[60] Nabbi A, Riabowol K. Rapid Isolation of Nuclei from Cells In Vitro. Cold Spring Harb Protoc. 2015;2015: pdb.prot083733. doi: 10.1101/pdb.prot083733 26240403

[61] Connor MG, Camarasa TMN, Patey E, Rasid O, Barrio L, Weight CM, et al. The histone demethylase KDM6B fine-tunes the host response to Streptococcus pneumoniae. Nat Microbiol. 2021;6: 257–269. doi: 10.1038/s41564-020-00805-8 33349663

[62] Jeong J-Y, Yim H-S, Ryu J-Y, Lee HS, Lee J-H, Seen D-S, et al. One-step sequence- and ligation-independent cloning as a rapid and versatile cloning method for functional genomics studies. Appl Environ Microbiol. 2012;78: 5440–3. doi: 10.1128/AEM.00844-12 22610439 PMC3416421

[63] Liu Y, Wang DL, Chen S, Zhao L, Sun FL. Oncogene Ras/phosphatidylinositol 3-kinase signaling targets histone H3 acetylation at lysine 56. J Biol Chem. 2012;287: 41469–41480. doi: 10.1074/jbc.M112.367847 22982396 PMC3510844

[64] C, Tan W, Whittle C, Qiu L, Cao L, Akbarian S, et al. The C-terminal TDP-43 fragments have a high aggregation propensity and harm neurons by a dominant-negative mechanism. PLoS One. 2010;5: e15878. doi: 10.1371/journal.pone.0015878 21209826 PMC3013128

[65] Britton S, Dernoncourt E, Delteil C, Froment C, Schiltz O, Salles B, et al. DNA damage triggers SAF-A and RNA biogenesis factors exclusion from chromatin coupled to R-loops removal. Nucleic Acids Res. 2014;42: 9047–9062. doi: 10.1093/nar/gku601 25030905 PMC4132723

[66] Shenoy AR, Visweswariah SS. Site-directed mutagenesis using a single mutagenic oligonucleotide and DpnI digestion of template DNA. Anal Biochem. 2003;319: 335–336. doi: 10.1016/s0003-2697(03)00286-0 12871732

