# Supplementary figures and images for: Histone H3 deacetylation promotes host cell viability for efficient infection by Listeria monocytogenes
Source: PLoS Pathog. 2021 Dec 20;17(12):e1010173. doi: 10.1371/journal.ppat.1010173 (PMC8722725; doi:10.1371/journal.ppat.1010173)

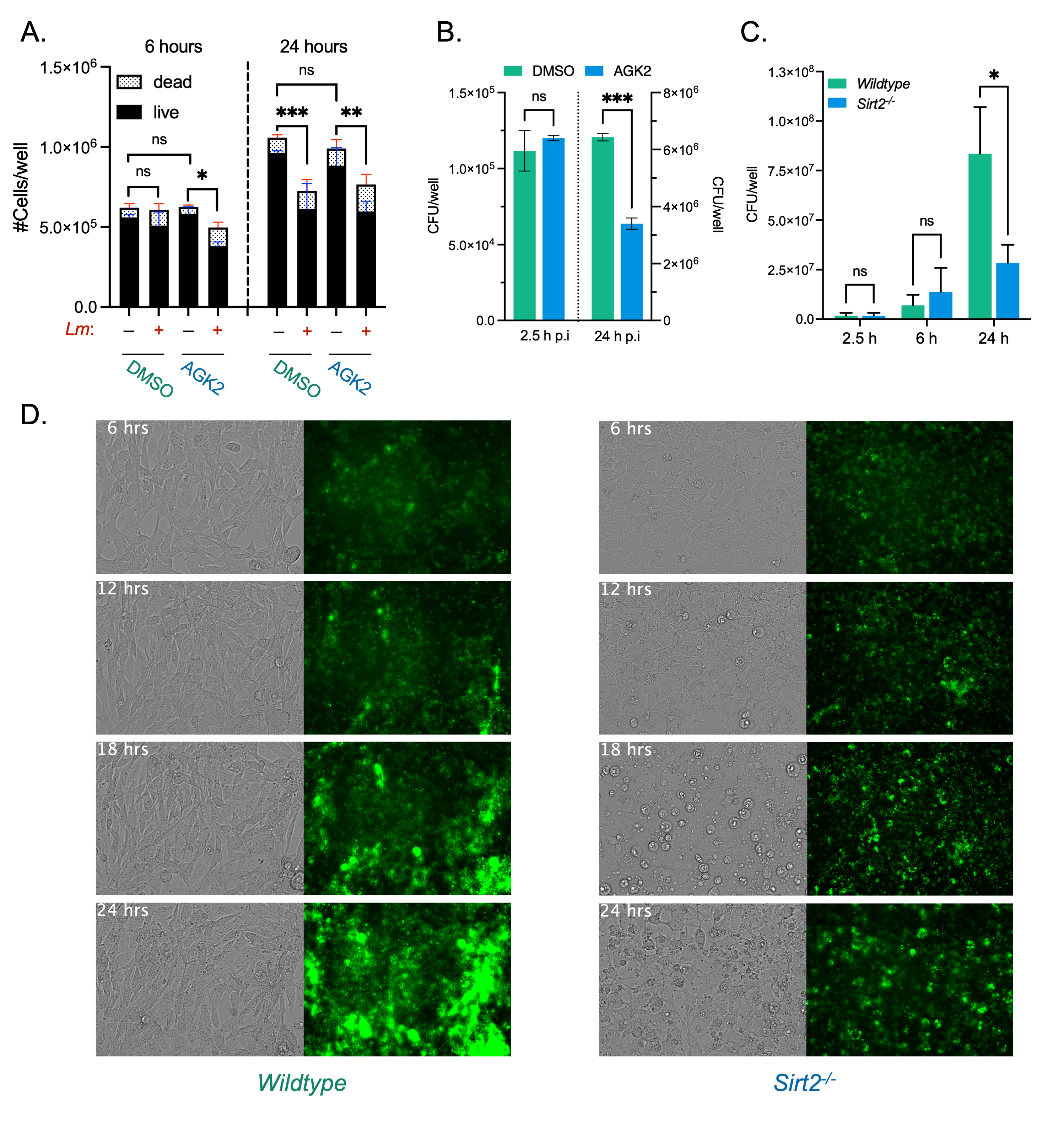

Supplement: S1 Fig — (A) Enumeration of live (Trypan negative) and dead (Trypan positive) HeLa cells pre-treated for 2 hours with DMSO or 5 mM AGK2 and infected for stated times. Cells were enumerated with Countess II Automated Cell Counter from 2 independent experiments. Statistical significance of live cells was determined by two-way ANOVA with FDR Benjamini-Hochberg (BH) correction for multiple comparisons (ns = not significant, * = p < 0.05, ** = p < 0.001), *** = p < <0.0001). (B) Quantification of L. monocytogenes intracellular CFUs from HeLa cells infected for 2.5 h or 24 h. Data are presented as CFU/well. Graphs display mean CFU ± SEM from 2 independent experiments. Statistical significance was determined by one-way ANOVA with FDR Benjamini-Hochberg (BH) correction for multiple comparisons (ns = not significant, *** = p < 0.001). (C) Quantification of L. monocytogenes intracellular CFUs from MEFs infected for 2.5 h, 6 h or 24 h. Data are presented as CFU/well. Graphs display mean CFU ± SEM from 2 independent experiments. Statistical significance was determined by one-way ANOVA with FDR Benjamini-Hochberg (BH) correction for multiple comparisons (ns = not significant, * = p < <0.05). (D) Representative images from S1 Movie. (TIF) [file ppat.1010173.s001.tif]

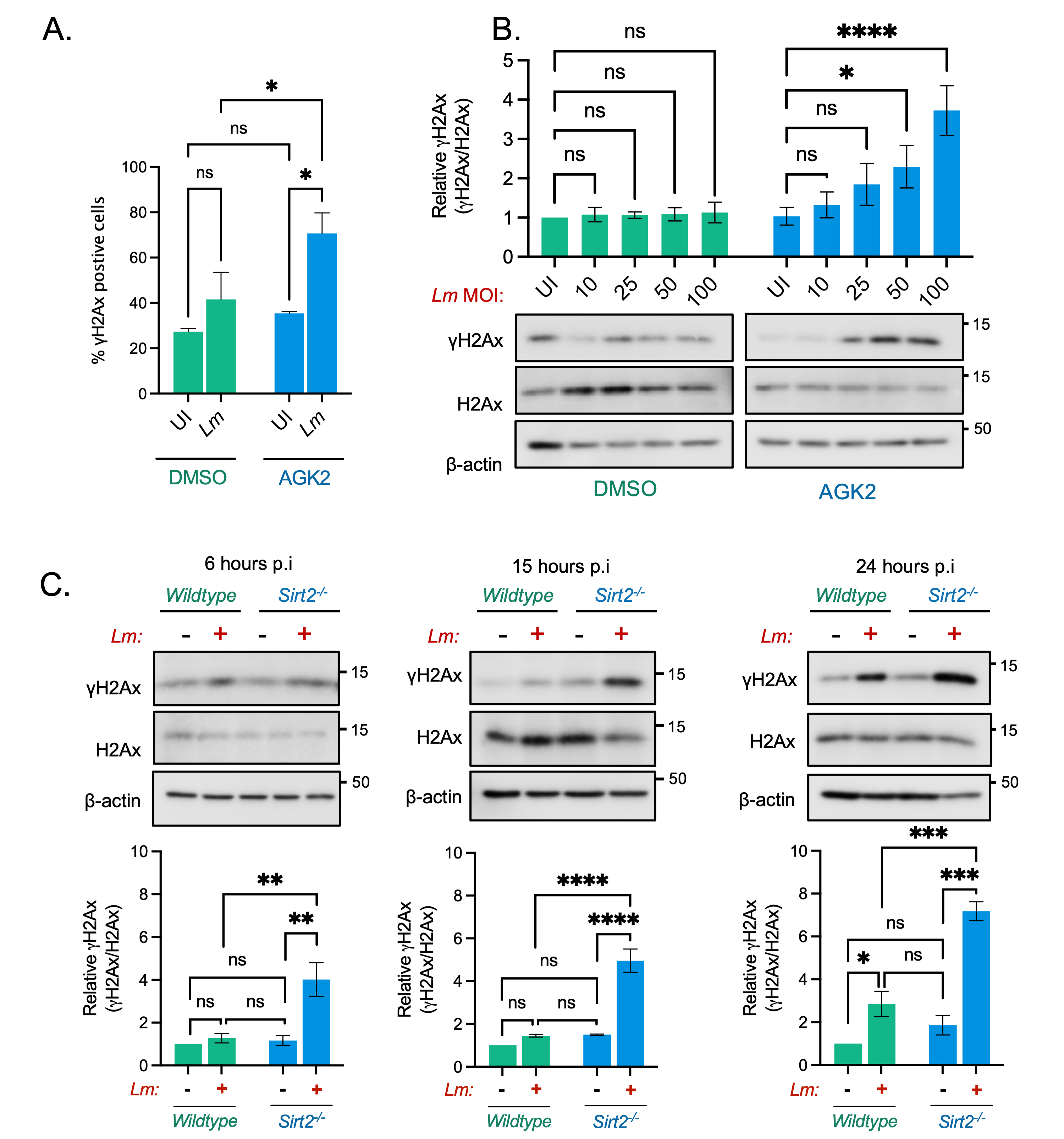

Supplement: S2 Fig — (A) Percentage of γH2Ax positive cells from Fig 1B. Error bars represent the SEM from four independent experiments. Statistical significance was calculated by two-way ANOVA with FDR Benjamini-Hochberg (BH) correction for multiple comparisons (ns = not significant, * = p < 0.05). (B) Immunoblot detection of γH2Ax and total H2Ax (left) from whole cell lysates of HeLa cells left uninfected (-) or infected with increasing MOIs of L. monocytogenes (Lm MOI) for 24 hours. (Above) Quantification of γH2Ax levels relative to uninfected. (C) Immunoblot detection of γH2Ax and total H2Ax (above) from whole cell lysates of wildtype and Sirt2-/- MEF cells left uninfected (-) or infected with increasing L. monocytogenes for stated timepoints. (Below) Quantification of γH2Ax levels relative to uninfected. Graph shows the mean ± SEM from at least three independent experiments. Statistical significance was determined by one-way ANOVA with FDR Benjamini-Hochberg (BH) correction for multiple comparisons (ns = not significant, ** = p < <0.005, *** = p < <0.001), **** = p < <0.0001. (TIF) [file ppat.1010173.s002.tif]

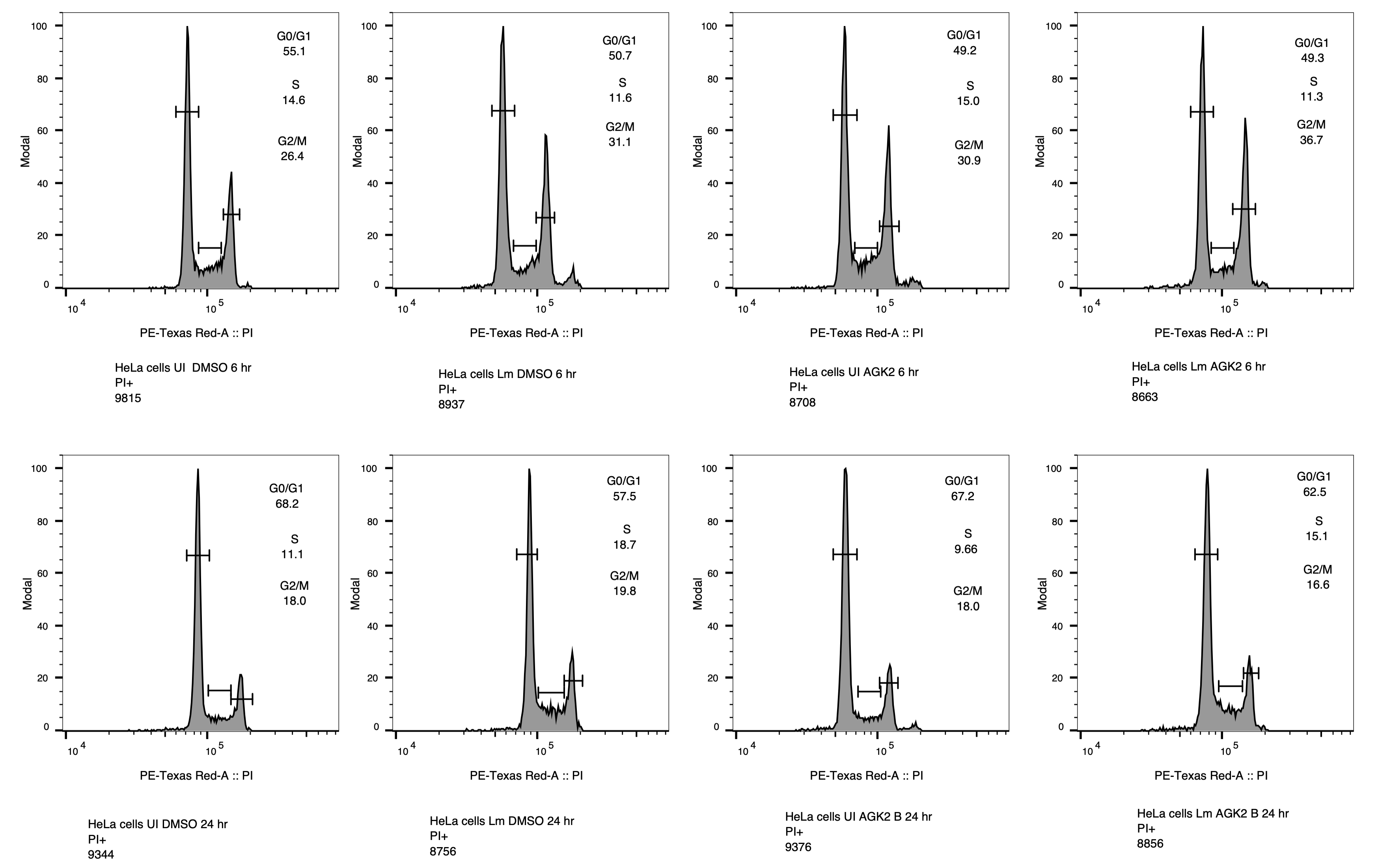

Supplement: S3 Fig — FACS analysis of propidium iodide-stained cells HeLa cells. Cells are untreated or treated with AGK2 then left uninfected or infected for stated times. Percentage of cells in each stage of the cell cycle is calculated with Flowjo software and presented with each histogram as % G0/G1, % S and % G2/M. Representative histograms of two independent experiments. (TIF) [file ppat.1010173.s003.tif]

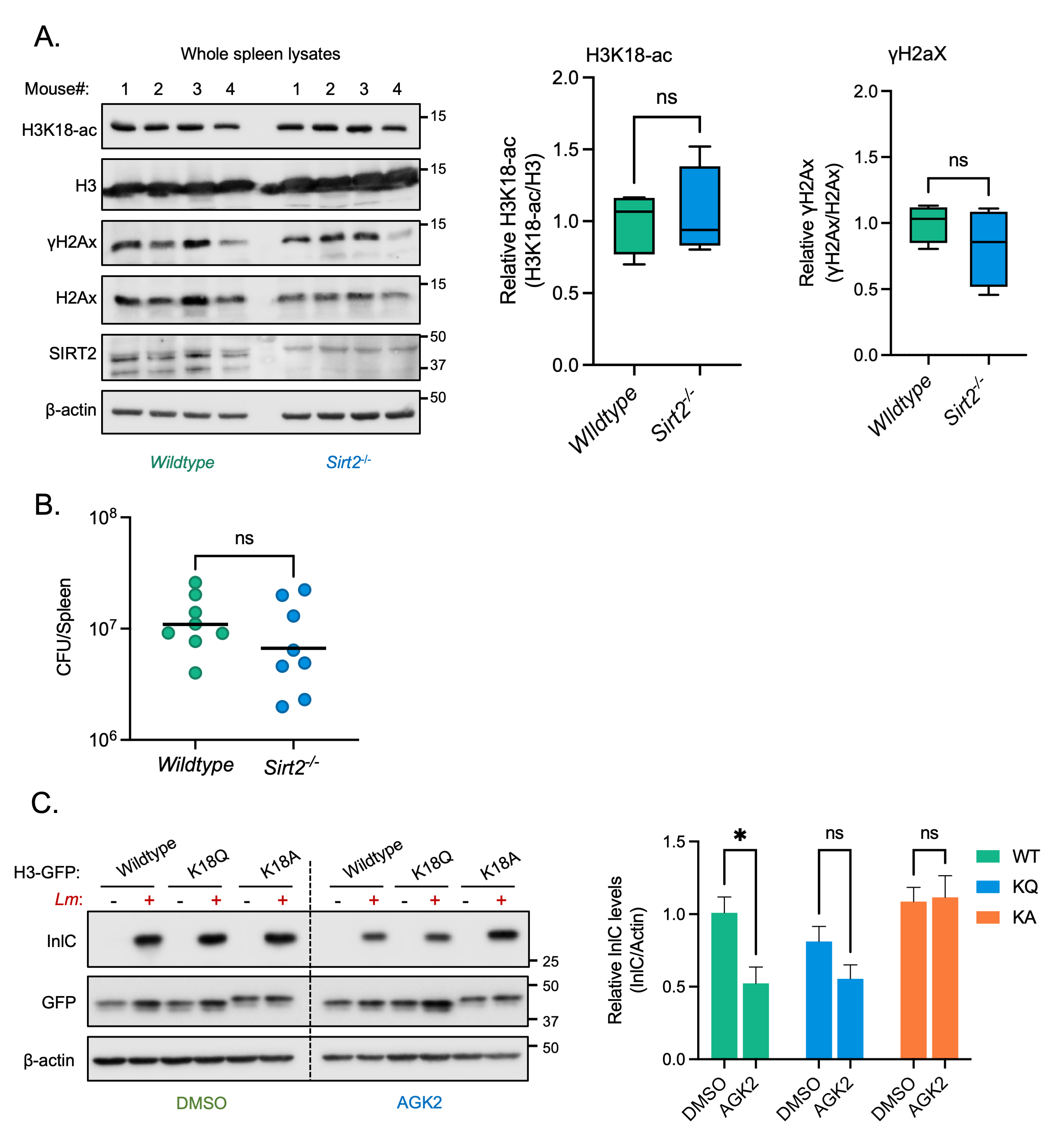

Supplement: S4 Fig — (A) Immunoblot detection of stated proteins from uninfected mouse spleen lysates (left). Quantification of normalised H3K18-ac and γH2Ax levels. Graphs show collated values from 4 mice, box and whisker plot with solid line denoting the median value. Statistical significance was determined by Two-tailed Unpaired t test (* = p < 0.05, *** = p <0.001). (B) Total L. monocytogenes CFU per spleen extracted from wildtype and Sirt2-/- mice 72 hours post infection. (C) Immunoblot detection of InlC (left) from whole cell lysates of HeLa cells left uninfected (-) or infected with L. monocytogenes (Lm) for 24 hours. Cells are expressing stated H3-GFP plasmids and treated with DMSO or 5 mM AGK2. Quantification of InlC levels (right) relative to uninfected. Results are expressed as intensity of actin normalised InlC. Graph shows the mean ± SEM from three independent experiments. Statistical significance was determined by two-way with FDR Benjamini-Hochberg (BH) correction for multiple comparisons (ns = not significant, * = p <0.05). (TIF) [file ppat.1010173.s004.tif]

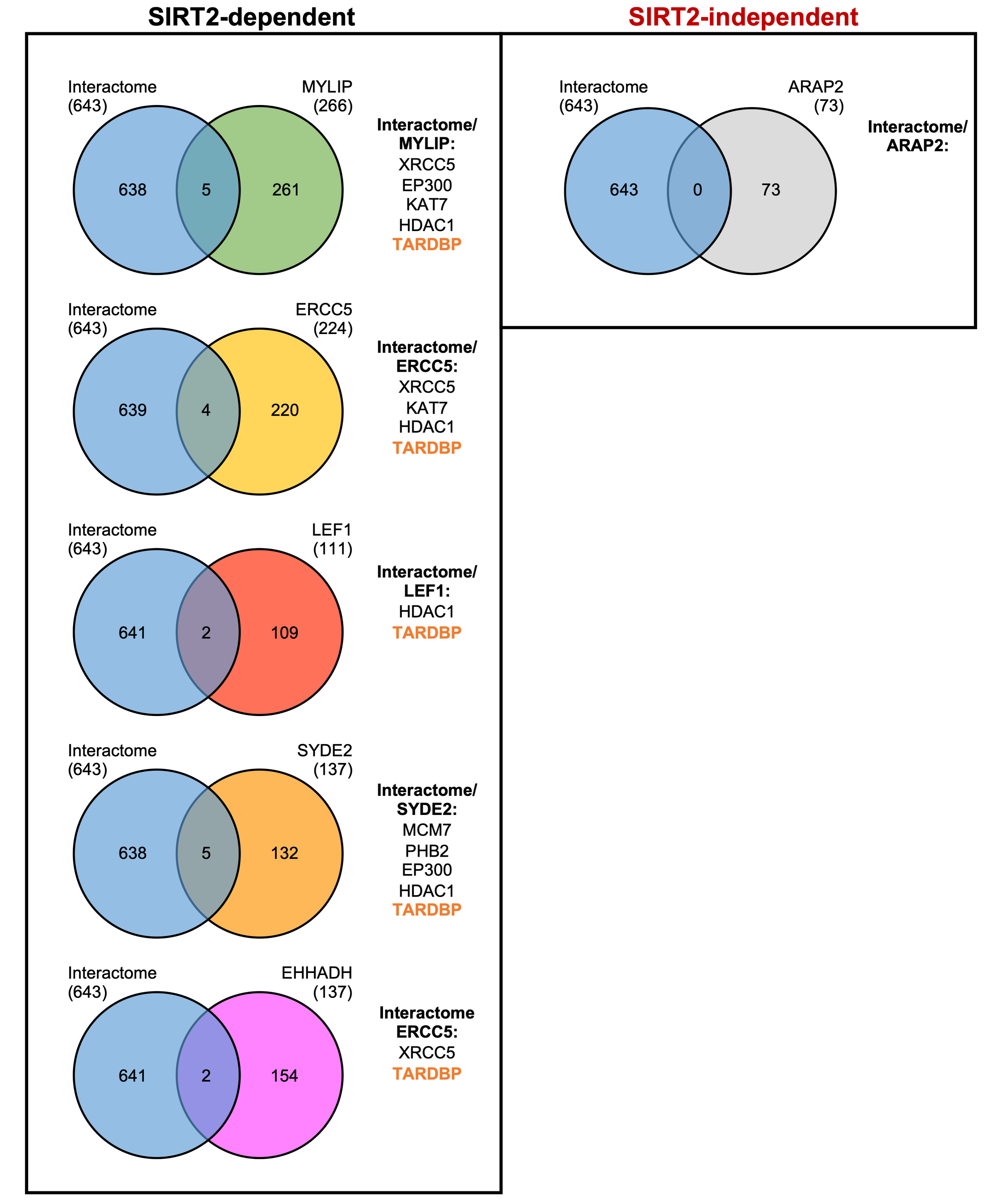

Supplement: S5 Fig — Venn diagrams illustrating proteins shared between SIRT2-interactome and interactors of the TSSs of MYLIP, ERRC5, LEF1, SYDE2, EHHADH and ARAP2. (TIF) [file ppat.1010173.s005.tif]

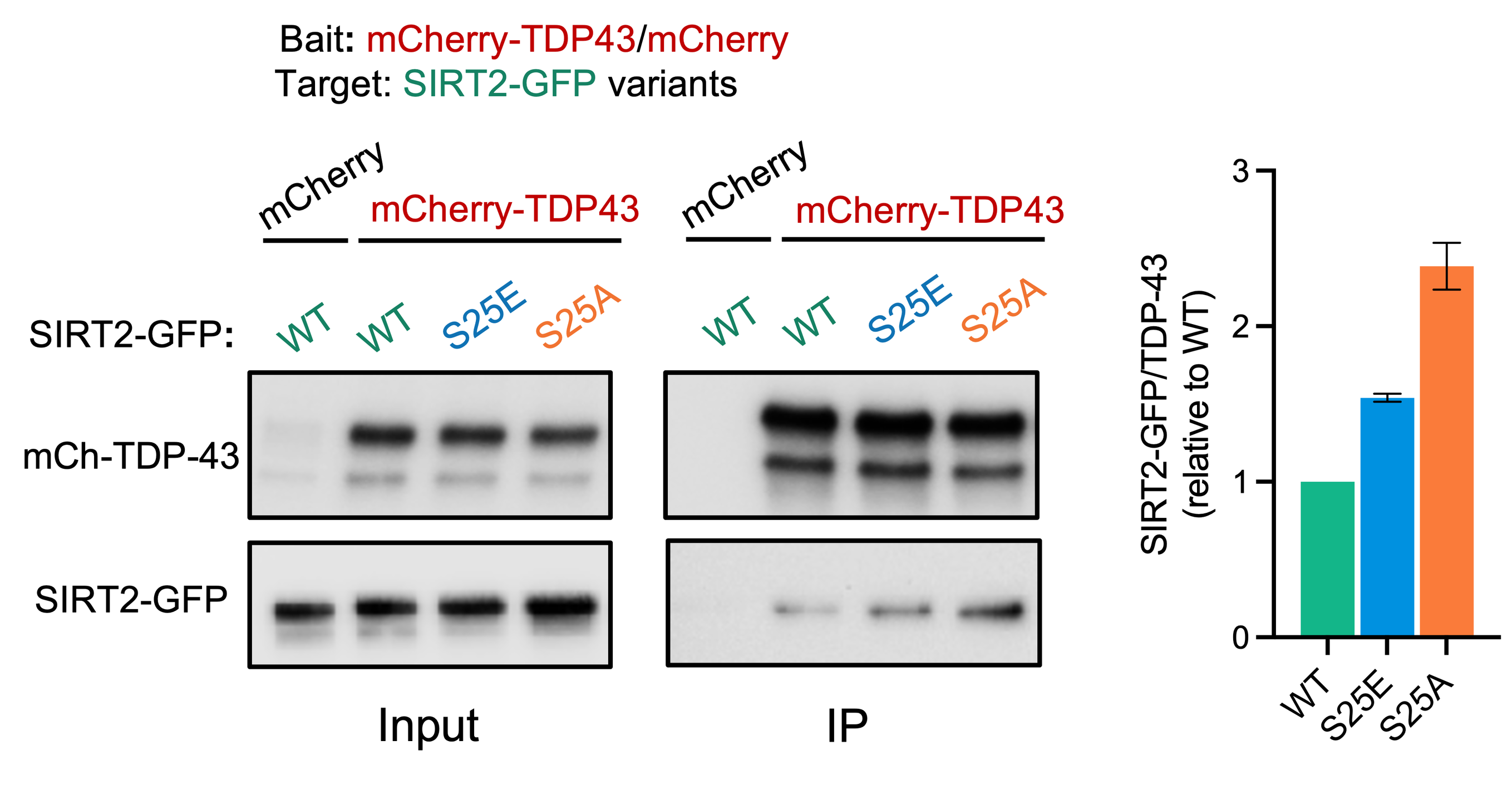

Supplement: S6 Fig — HeLa cells expressing either mCherry alone or mCherry-TDP-43 were co-transfected with stated variants of SIRT2-GFP followed by immunoprecipitation using RFP-Trap agarose beads. Cell lysates (Input) and IP fractions were immunoblotted using antibodies against TDP-43 or GFP for detection of SIRT2. (TIF) [file ppat.1010173.s006.tif]

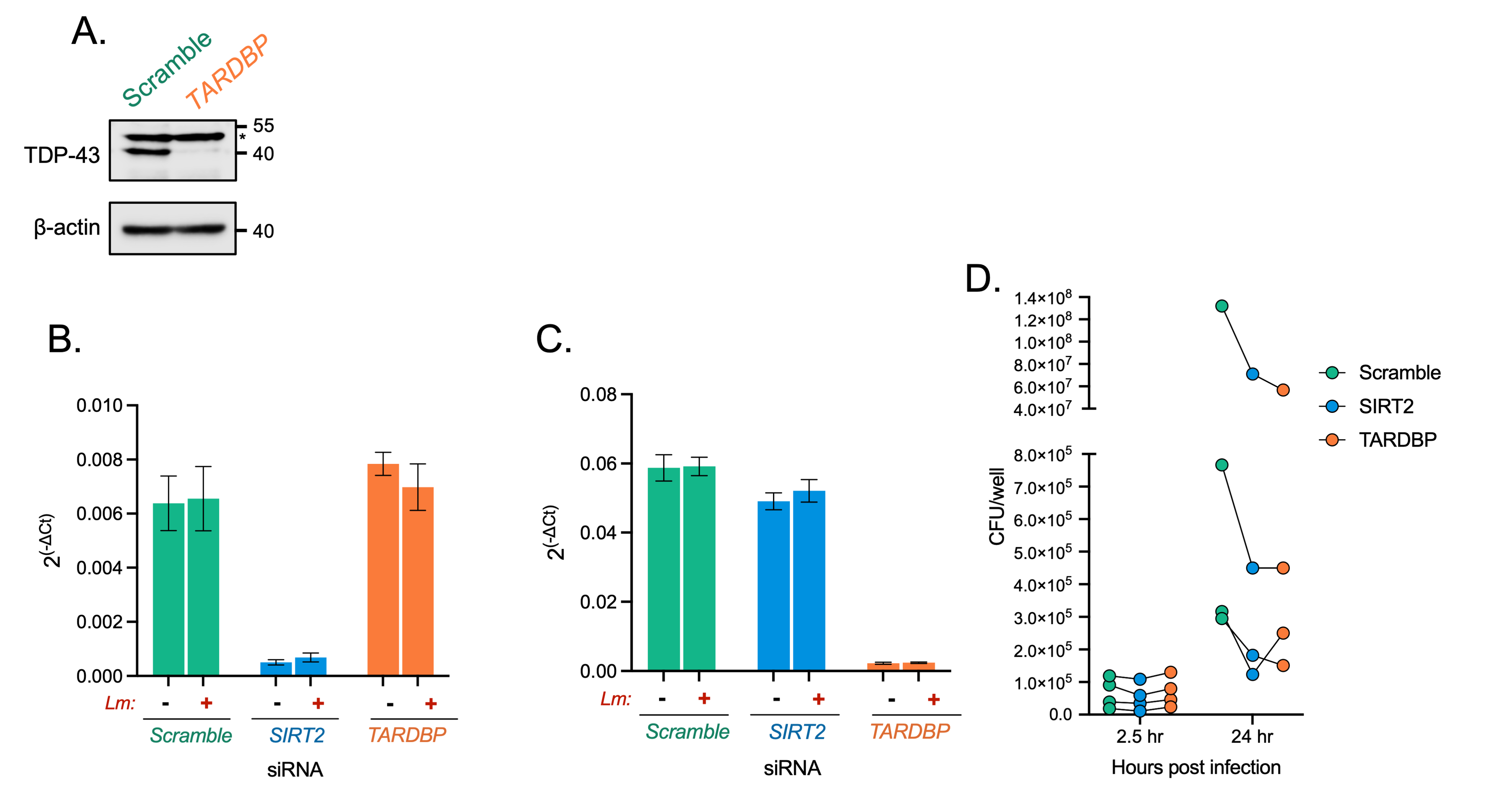

Supplement: S7 Fig — (A) Western blot showing TDP-43 knockdown following siRNA transfection for 48 hours. Relative mRNA expression of (B) SIRT2 and (C) TARDBP as detected by qPCR normalised to GAPDH. Mean ± S.E.M from three independent experiments are plotted. (D) Quantification of L. monocytogenes intracellular CFUs. HeLa cells were transfected with indicated siRNAs and infected for 2.5 h or 24 h. Lysates were plated onto BHI agar and bacterial CFUs were enumerated. Data are presented as CFU/well. Individual biological replicates are plotted as paired values. (TIF) [file ppat.1010173.s007.tif]

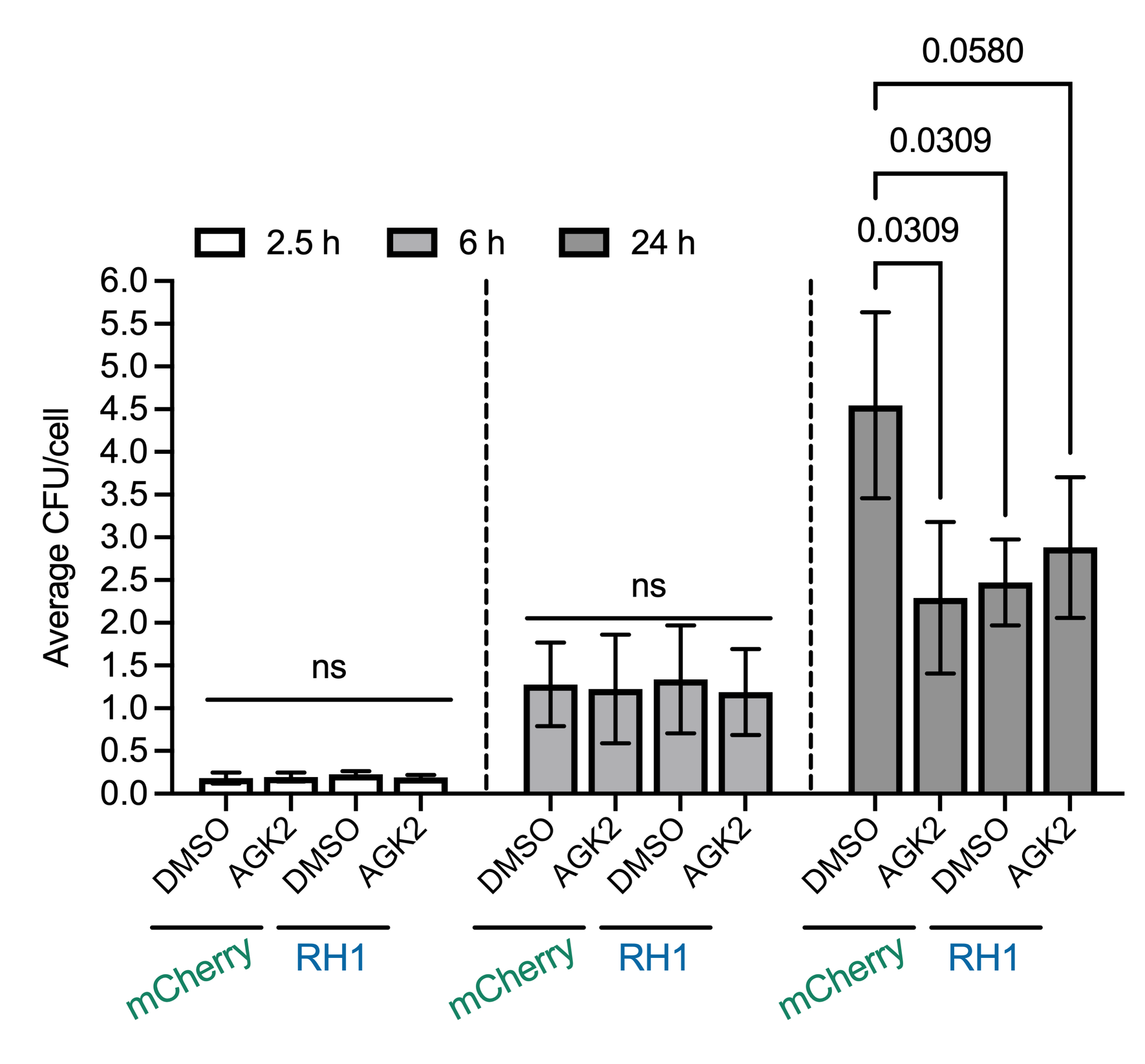

Supplement: S8 Fig — Quantification of L. monocytogenes intracellular CFU/cell. HeLa cells expressing either mCherry or RNaseH1 were treated with DMSO or 5 mM AGK2 then infected with L. monocytogenes. Intracellular bacteria were extracted at 2.5, 6 and 24 hours post infection plated onto BHI agar and bacterial CFUs were enumerated. Data are presented as average CFU/cell. Mean ± S.E.M from three independent experiments are plotted. (TIF) [file ppat.1010173.s008.tif]

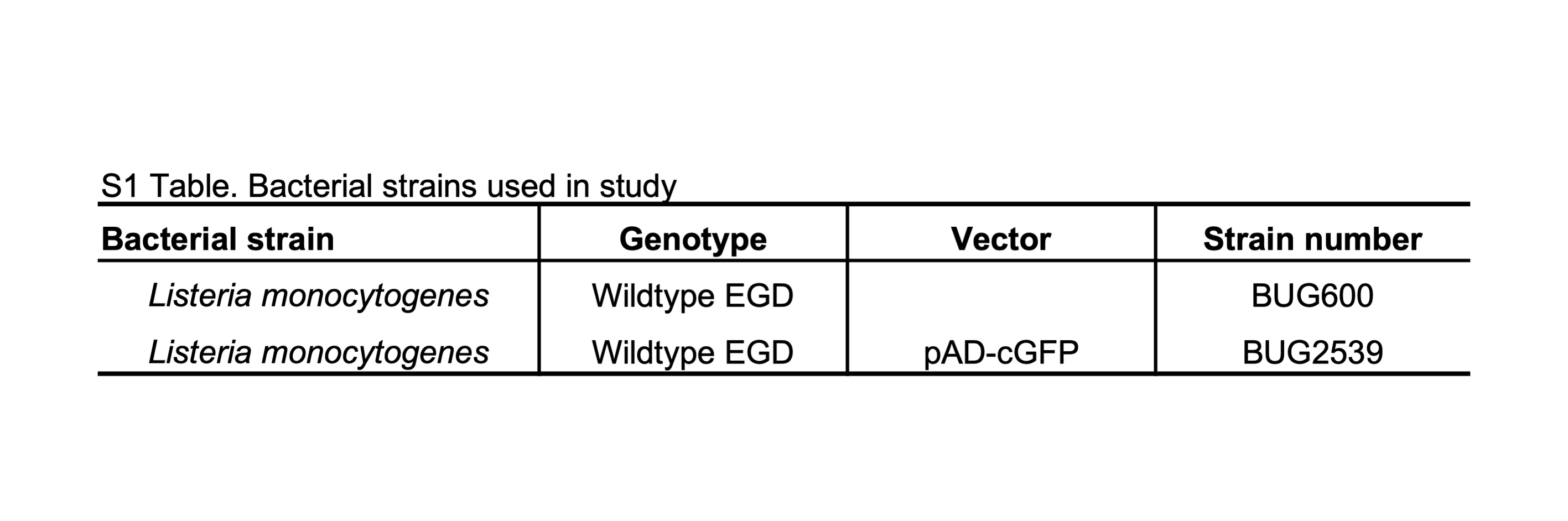

Supplement: S1 Table — (TIF) [file ppat.1010173.s010.tif]

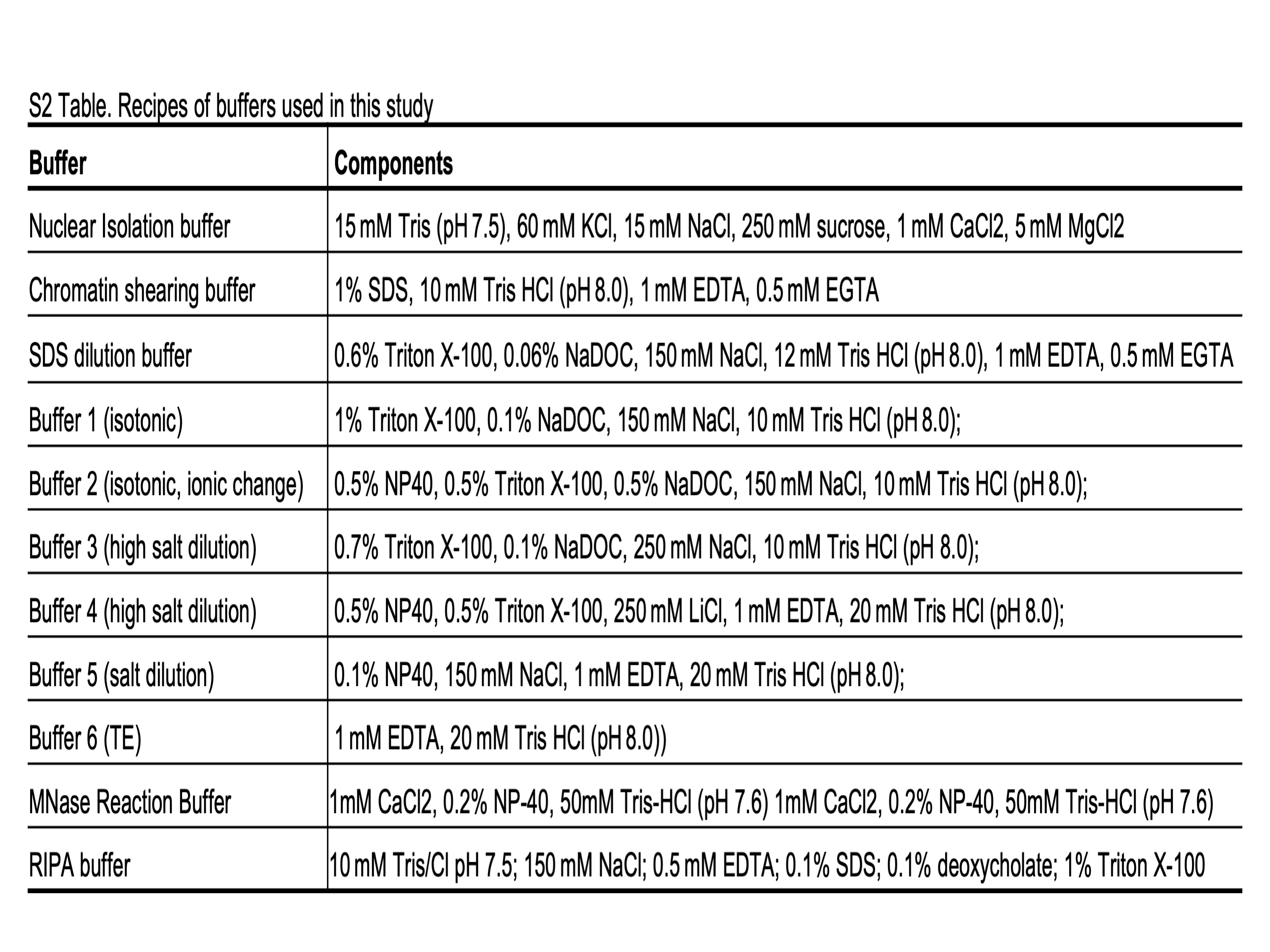

Supplement: S2 Table — (TIF) [file ppat.1010173.s011.tif]

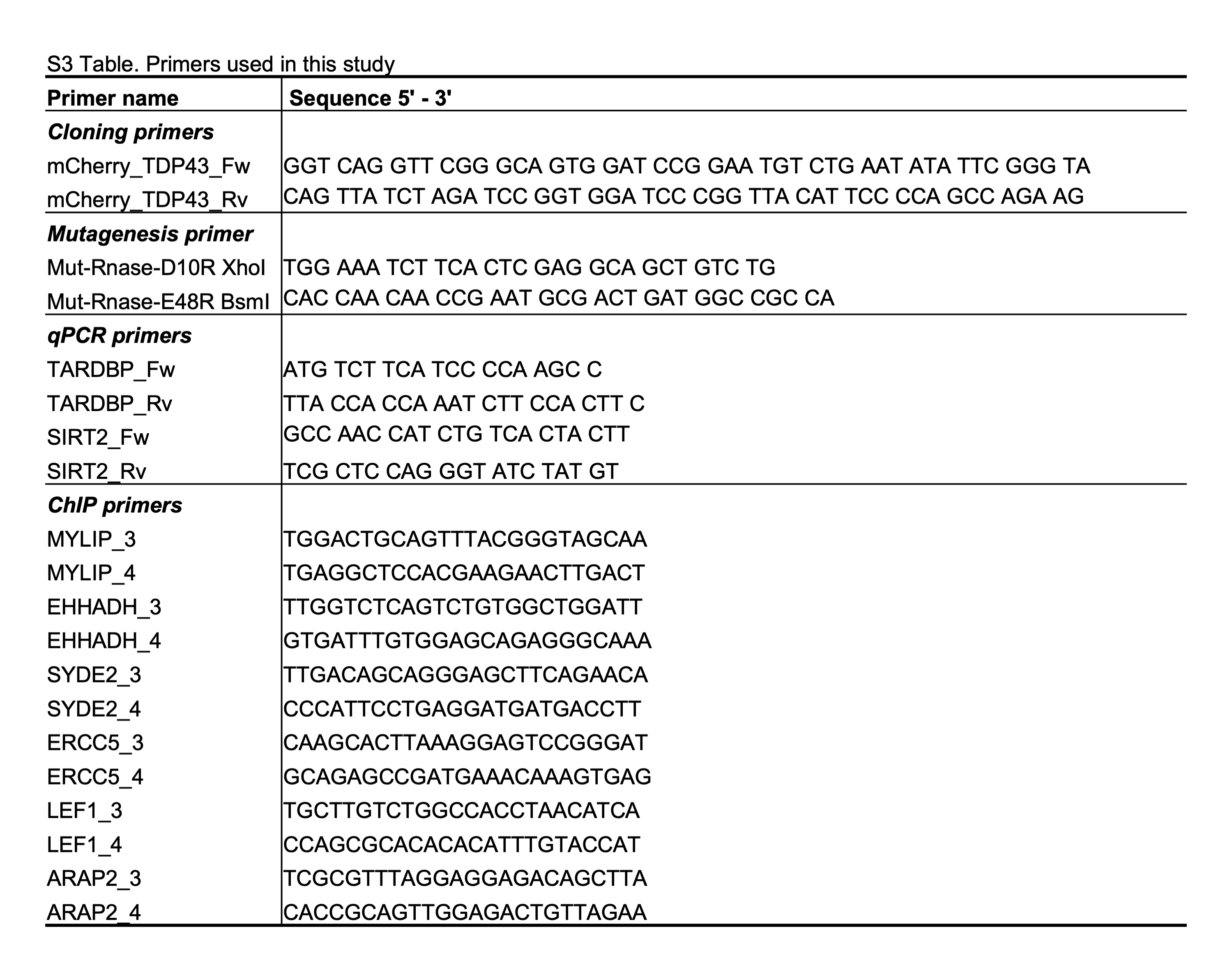

Supplement: S3 Table — (TIF) [file ppat.1010173.s012.tif]

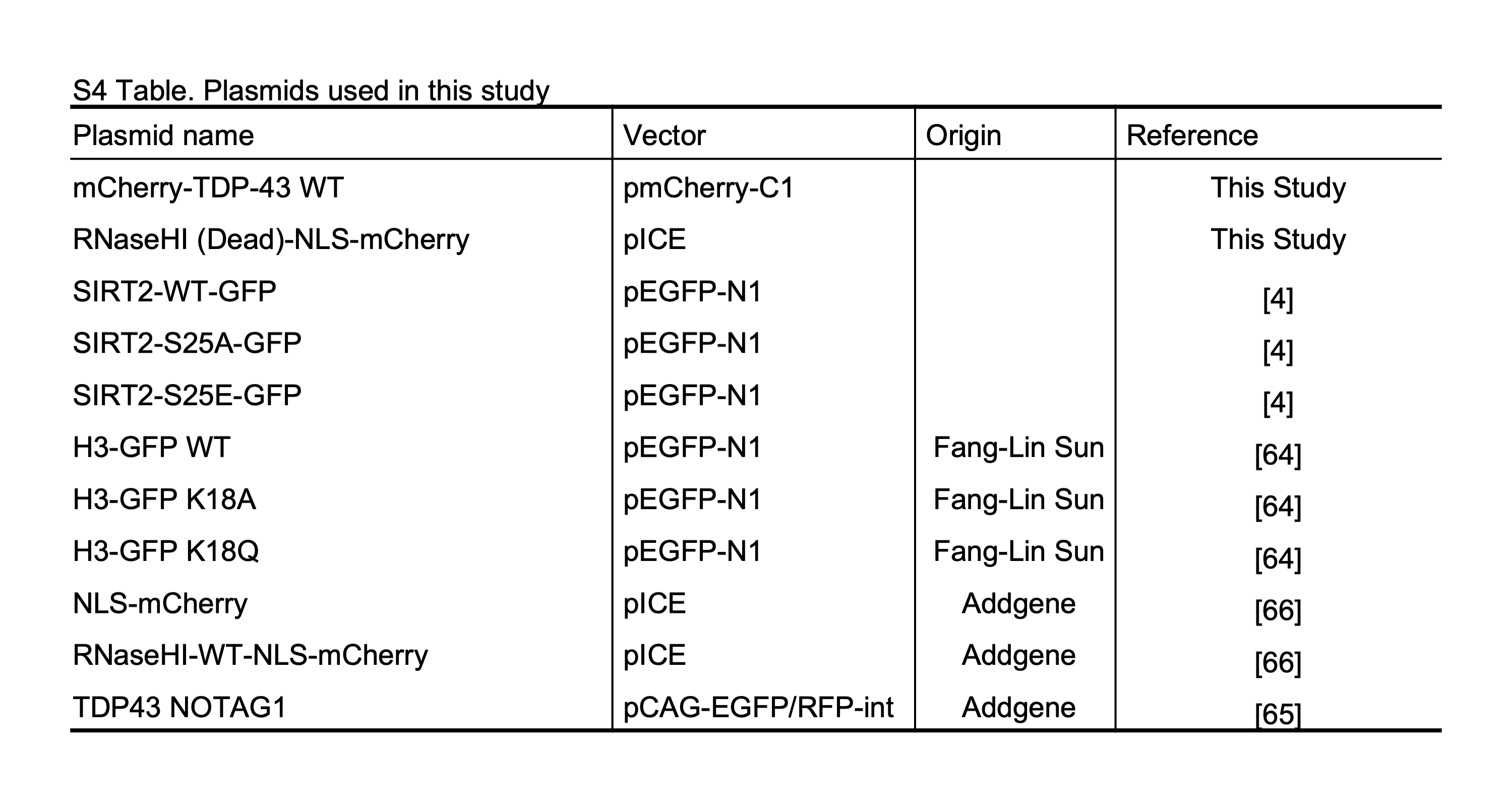

Supplement: S4 Table — (TIF) [file ppat.1010173.s013.tif]
